# Facile synthesis of Gd/Ru-doped fluorescent carbon dots for fluorescent/MR bimodal imaging and tumor therapy

**DOI:** 10.1186/s12951-024-02360-4

**Published:** 2024-03-02

**Authors:** Yupeng Shi, Yaning Xia, Mengyang Zhou, Yifei Wang, Jianfeng Bao, Yong Zhang, Jingliang Cheng

**Affiliations:** Department of MRI, Henan Key Laboratory of Functional Magnetic Resonance Imaging and Molecular Imaging, The First Affiliated Hospital of Zhengzhou University, Zhengzhou, 450052 China

**Keywords:** Carbon dots, MR imaging, Red fluorescence, Tumor imaging, PDT

## Abstract

Functional metal doping endows fluorescent carbon dots with richer physical and chemical properties, greatly expanding their potential in the biomedical field. Nonetheless, fabricating carbon dots with integrated functionality for diagnostic and therapeutic modalities remains challenging. Herein, we develop a simple strategy to prepare Gd/Ru bimetallic doped fluorescent carbon dots (Gd/Ru-CDs) via a one-step microwave-assisted method with Ru(dcbpy)_3_Cl_2_, citric acid, polyethyleneimine, and GdCl_3_ as precursors. Multiple techniques were employed to characterize the morphology and properties of the obtained carbon dots. The Gd/Ru-CDs are high mono-dispersity, uniform spherical nanoparticles with an average diameter of 4.2 nm. Moreover, X-ray photoelectron spectroscopy (XPS), and Fourier transform infrared (FTIR) confirmed the composition and surface properties of the carbon dots. In particular, the successful doping of Gd/Ru enables the carbon dots not only show considerable magnetic resonance imaging (MRI) performance but also obtain better fluorescence (FL) properties, especially in the red emission area. More impressively, it has low cytotoxicity, excellent biocompatibility, and efficient reactive oxygen species (ROS) generation ability, making it an effective imaging-guided tumor treatment reagent. In vivo experiments have revealed that Gd/Ru-CDs can achieve light-induced tumor suppression and non-invasive fluorescence/magnetic resonance bimodal imaging reagents to monitor the treatment process of mouse tumor models. Thus, this simple and efficient carbon dot manufacturing strategy by doping functional metals has expanded avenues for the development and application of multifunctional all-in-one theranostics.

**Supplementary Information:**

The online version contains supplementary material available at 10.1186/s12951-024-02360-4.

## Introduction

Over recent decades, carbon dots have emerged as potent nanomaterials in the field of biomedicine, attributed to their superior optical characteristics, robust stability, and high degree of biocompatibility [1–4]. These attributes facilitate their deployment in a myriad of biomedical applications encompassing bioimaging, biosensing, drug delivery, and nanomedicine [5–7]. Initial research primarily focused on synthesizing carbon dots with a fluorescence emission range from blue to orange under the stimulus of ultraviolet or blue light [3, 8]. Yet, the narrow spectrum of excitation and emission confined the in vivo applicability of these carbon dots for optical imaging, invoking concerns such as potential harm to living organisms and restricted tissue penetration depth, alongside interference from the innate autofluorescence of biological tissues. Acknowledging these limitations, the past decade witnessed concerted efforts to steer the fluorescence emission properties of carbon dots towards the red to near-infrared (NIR) range, principally through the process of doping [9–12]. This innovation incorporated elements like boron, nitrogen, and sulfur into the carbon dot matrix [13, 14]. Numerous research collectives have reported the creation of red fluorescent carbon dots, with a subset achieving successful deployment in animal imaging endeavors [15]. Despite this progress, a solitary optical imaging mode remains insufficient to satisfy the pressing demands of precise cancer diagnostics. Consequently, recent years have seen a surge in interest in multimodal imaging, which amalgamates two or more imaging techniques into a singular nanoprobe, including but not limited to optical imaging, MR imaging, ultrasound, positron emission tomography (PET), computed tomography (CT), and photoacoustic (PA) imaging approaches [16–19]. This multi-faceted approach garners a wealth of tissue data, leveraging the distinctive benefits of each modality to enhance the reliability and accuracy of disease site detection. Predominantly, nanoprobes that integrate optical and magnetic resonance imaging functionalities have gained prominence. MR imaging stands as a formidable diagnostic tool, enabling the non-invasive acquisition of high-resolution, detailed anatomical images, coupled with extensive tissue penetration capabilities [20, 21]. Contrastingly, optical imaging offers high molecular sensitivity and facilitates swift screenings, carving out its niche as a non-destructive technique [22]. Thus, marrying carbon dot platforms with MR imaging augments their utility significantly, promising a rich tapestry of high-resolution images laden with intricate anatomical or biological insights pertaining to the target area, thereby elevating the prospects of optical imaging applications [23, 24].

Recently, nanotheranostics has arisen as a potent approach to cancer treatment, spearheading the development of innovative, multifunctional nanostructured materials that amalgamate imaging and therapeutic functionalities [25–27]. These intricate nanoplatforms are devised by harmonizing the aforementioned imaging techniques with a diverse arsenal of therapeutic agents such as chemotherapeutic drugs, radiosensitizers, photosensitizers, and immune adjuvants. This theranostics paradigm paves the way for concurrent diagnosis and targeted tumor eradication with a singular dose, facilitating image-guided cancer interventions [28]. Photodynamic therapy (PDT) positions itself as an effective, non-invasive treatment modality, leveraging photosensitizers to generate singlet oxygen and other reactive oxygen species, thereby achieving focused tumor ablation [29, 30]. While the conventional roster of photosensitizers encompasses entities like porphyrins, phthalocyanines, and ruthenium bipyridyl metal complexes, they are frequently marred by high toxicity, inadequate water solubility, and compromised stability, thereby undermining their therapeutic efficacy [31]. This underscores the imperative of engendering novel photosensitizers. A diverse array of nanomaterials, including metal framework substances and carbon nanomaterials, have been extensively explored as viable PDT agents [32]. Carbon dots (CDs) have also been recently documented as proficient PDT agents in the oncological treatment landscape [33, 34]. Illustratively, Ge et al. pioneered the utilization of porphyrin-derived carbon sources to craft near-infrared responsive nitrogen-doped carbon dots, facilitating effective photodynamic therapy in tumor settings [20].

**Scheme 1:**
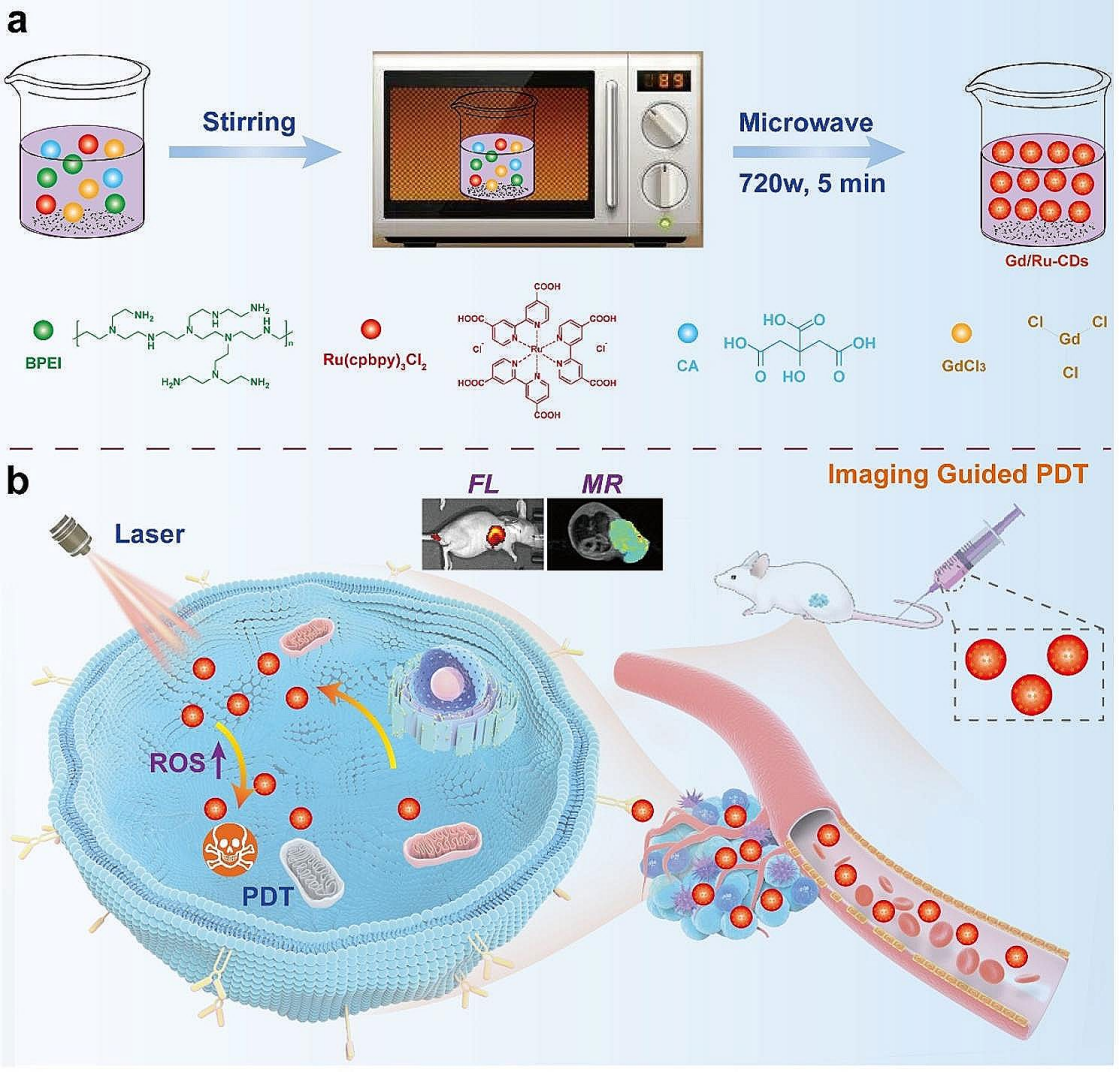
Schematic diagram of microwave-assisted synthesis of Gd/Ru-CDs (**a**) and (**b**) FL/MR-guided photodynamic therapy of tumor

In the current investigation delineated in Scheme 1, we embarked on the synthesis of gadolinium/ruthenium-doped carbon dots (Gd/Ru-CDs) through a one-step microwave-assisted synthesis technique, earmarked for FL/MR imaging-guided photodynamic cancer therapy. A meticulous assessment of the Gd/Ru-CDs encompassed the evaluation of their morphological, physicochemical, optical attributes, coupled with an exploration of their MR contrast and photodynamic properties. Capitalizing on these favorable traits, Gd/Ru-CDs demonstrated prowess in fluorescence and MR dual-modal tumor imaging, both in vitro and in vivo. Further, the study spotlighted the efficacy of imaging-guided PDT, showcasing pronounced anticancer potency in 4T1 tumor-bearing mice. The investigation culminated with the affirmation of the outstanding biocompatibility of Gd/Ru-CDs, corroborated through in vitro and in vivo assays. This portfolio of attributes cements the Gd/Ru-CDs as stellar candidates for FL/MR dual-modal imaging nanoprobes, holding substantial promise as PDT agents in cancer diagnostic and therapeutic realms.

## Experiments

### Materials

All materials and reagents were used without purification unless otherwise stated. Citric acid, gadolinium chloride and Tris(4,4’-dicarboxylicacid-2,2’-bipyridyl)ruthenium(II) dichloride (Ru(dcbpy)_3_Cl_2_) were purchased from Aladdin Technology Co., Ltd. 2’,7’-Dichlorodihydrofluorescein diacetate (DCFH-DA) obtained from Sigma. Dulbecco’s Modified Eagle Medium (DMEM), RPMI-1640 Medium, and fetal bovine serum (FBS) were purchased from Sigma-Aldrich. All solutions were prepared using deionized water produced by a Milli-Q water purification system (Millipore, USA) to a minimum resistivity of 18.2 MΩ.

### Characterization

The fluorescence emission spectra were recorded using a by Horiba fluorophotometer. Cell imaging experiments were carried out using a confocal microscope (Zeiss LSM-710, Zeiss, Germany). X-ray photoelectron spectroscopy (XPS) analysis of Gd/Ru-CDs was performed using an ESCALAB 250 spectrometer with a monochromatic X-ray source with Al Ka excitation (1486.6 eV). The Fourier transform infrared (FTIR) spectrum was measured on a Bruker Vector-22 FTIR spectrometer at room temperature. TEM images were captured by an HT 7800 transmission electron microscope (Tokyo, Japan). Diameter and ζ-potential were measured on a Malvern Nano-ZS particle sizer (Malvern, UK). UV-vis spectra were recorded using a UV-2700 spectrophotometer (Shimadzu, Japan). In vivo fluorescence imaging was carried out on an IVIS spectrum (PerkinElmer, USA).

### Synthesis of Gd/Ru-CDs

In this study, undoped CDs and Gd/Ru-CDs were prepared by microwave-assisted synthesis [35]. Briefly, take a 50 mL beaker, add 10 mL of distilled water, and add 1.00 g of branched PEI, 0.5 g citric acid, a certain amount of Ru(dcbpy)_3_Cl_2_, and GdCl_3_. Subsequently, the mixture was heated in a household microwave oven (750 W, Galanz, China) for 5 min. After cooling, redistilled water was added to dissolve the formed Gd/Ru-CDs, and the solution was collected and centrifuged at 6000 rpm for 10 min. Then, the supernatant was collected and dialyzed for 24 h with a cellulose ester membrane dialysis bag with a molecular weight cut-off of Mw = 1400, and then freeze-dried to obtain Gd/Ru-CDs powder for further use.

### Cytotoxicity assay

4T1 cells (provided by the Cell Bank of the Chinese Academy of Sciences) were cultured in an atmosphere of 5% CO_2_/95% air at 37 °C, supplemented with 10% FBS, penicillin (50 U mL^− 1^), streptomycin (0.05 mg mL^− 1^) DMEM (high glucose) medium. The CCK-8 kit was used to evaluate the cytotoxicity of different concentrations of Gd/Ru-CDs. Briefly, Gd/Ru-CDs at concentrations ranging from 0 to 1.0 mM were co-incubated with breast cancer 4T1 cells for 24 h, and then the medium was discarded and washed twice with PBS. Then CCK-8 reagent was added and incubated for another 4 h, followed by colorimetric analysis with a microplate reader. Each experiment was performed in triplicate.

### Cell imaging

For cell imaging assay, 4T1 cells were seeded on 6 mm glass coverslips (1 × 10^5^ cells per slip) and allowed to adhere for 24 h, followed by the treatment with 0.2 mg mL^− 1^ Gd/Ru-CDs suspension for 4 h. Then, the cells were washed several times with PBS (pH = 7.4), The cells were then stained with Hoechst 3342 (10 μg mL^− 1^) for 10 min at 37 °C, respectively. After removal of the medium, the cells were observed through the imaging system.

### Reactive oxygen species (ROS) generation

In vitro PDT capacity investigation ^1^O_2_ generation was determined by 9,10-anthracenediyl-bi(methylene) dimalonic acid (ABDA) assay. Gd/Ru-CDs (50 μg mL^− 1^) was mixed with ABDA (100 μM), then the mixture was irradiated by an LED blue light (0.1 W cm-^2^) lamp. The absorption of ABDA at 380 nm was monitered every five mins. Gd/Ru-CDs (50 μg mL^−^) and ABDA (100 μM) at the same concentration were set as controls. Intracellular ROS production of Gd/Ru-CDs by DCFH-DA with 4T1 cells. Briefly, 4T1 cells were cultured with Gd/Ru-CDs in a confocal dish at 37 °C for 24 h. Then the cells containing Gd/Ru-CDs were incubated with DCFH-DA (10 μM) for another 20 min. After irradiation by white light, the intracellular ROS generation was performed by confocal laser scanning microscopy.

### In vitro MR imaging

The longitudinal relaxation time (T1) of nanoprobe serial dilutions was measured on a clinical 3.0T MRI instrument. Apply a standard inversion recovery pulse sequence and use pure water as control. T1-weighted MR images of serial dilutions of Gd/Ru-CDs were acquired using a Siemens 3.0T skyro body coil, applying a standard spin echo sequence, 550 ms TR and 15 ms TE.

### In vivo fluorescence imaging

4–6 weeks-old female BALB/c nude mice were selected as animal models. The Ethics Committee approved the animal experiments of the Animal Experimental Center of Zhengzhou University. When the tumor grew to nearly 100 mm^3^, Gd/Ru-CDs were injected through the tail vein. The fluorescence intensity of the tumor site was measured at 0, 2, 4, 6, 8, 12, and 24 h after injection with a living animal imager.

### In vivo MR imaging

First, tumor model mice were built by injecting 100 μL of 4T1 cells (5 × 10^6^ cells/mL). After 14 days, the volume was 100 mm^3^, and a SIEMENS Skyra 3.0 T MR scanner was employed to evaluate the in vivo MR imaging performance of Gd/Ru-CDs. Before injection, the T1-weighted MR imaging of the tumor site was recorded. Then, the mouse was injected with 100 μL of Gd/Ru-CDs solution (1 mg/mL) via a tail vein. After 0, 1, 2, 4, 8, and 24 h, the T1-weighted MR imaging was re-recorded.

### In vivo antitumor effect

In vivo photodynamic therapy was conducted on 4T1 tumor-bearing BALB/c mice. The mice were randomly distributed into four groups (6 mice in each group) for the in vivo experiments. The mice were intravenously injected with different formulations: (i) Saline, (ii) Saline + Laser (L), (iii) Gd/Ru-CDs, (v) Gd/Ru-CDs + L (5 mg/ kg). Then, 650 nm laser-irradiation (1 W/cm^2^, 20 min) was conducted after 3 h post-injection for some groups of mice at the tumor region. To monitor tumor progression, the tumor volume changes were measured, and the mice bodyweight was also recorded. After three weeks measurement, tumors were collected from the sacrificed mice after various treatments for further analysis.

### Statistical analysis

Each experiment was repeated three times, and the results were expressed as mean ± standard deviation. One-way ANOVA was used to compare the differences between the data groups. *P* < 0.05 indicated that the difference was statistically significant.

## Results and discussion

**Fig. 1:**
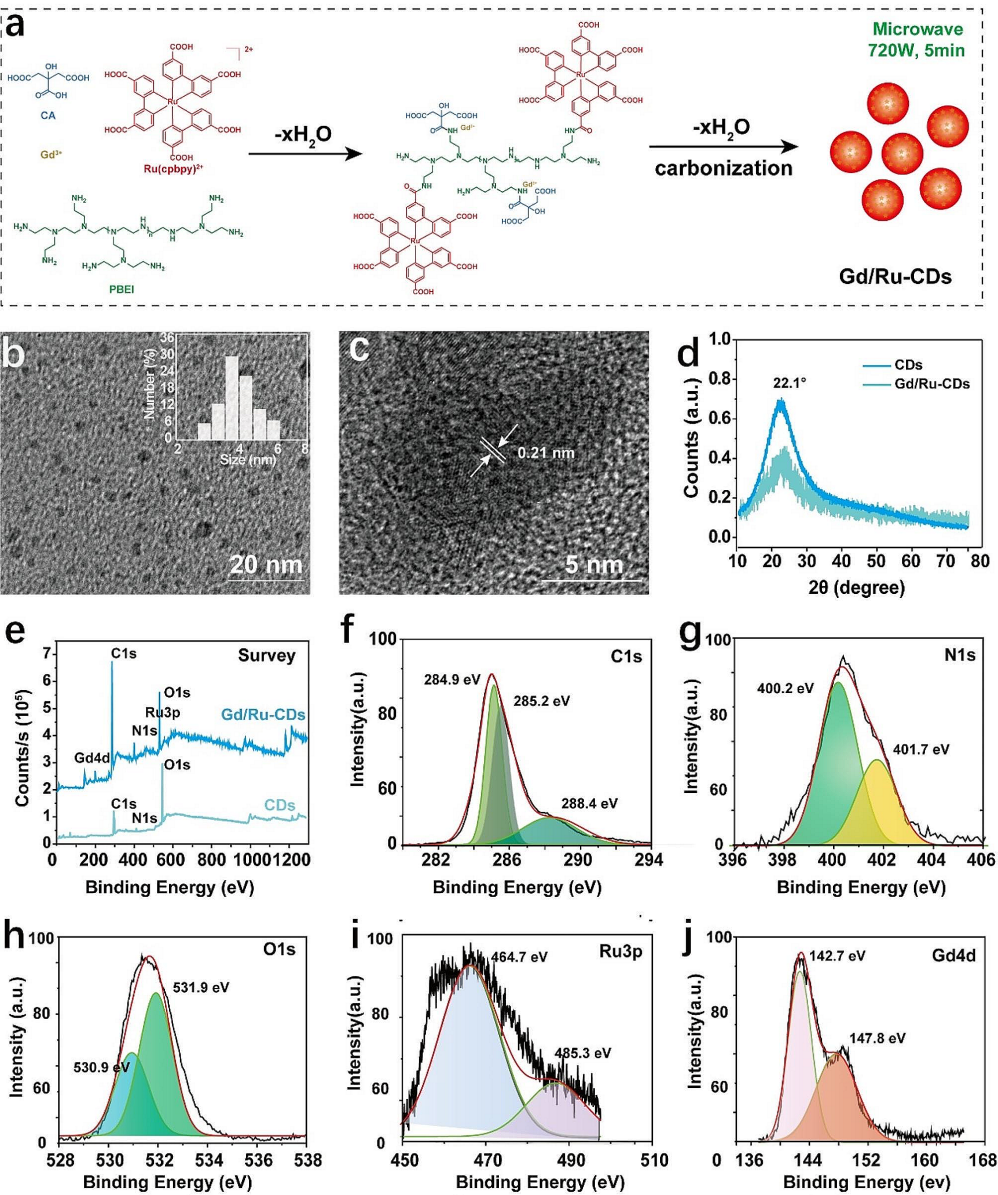
(**a**) The synthesis route of Gd/Ru-CDs by facile microwave methods in 5 min; (**b**) TEM image of Gd/Ru-CDs (insert image represent the size distribution); (**c**) HRTEM image of Gd/Ru-CDs, Lattice spacing: 0.21 nm; (**d**) XRD of CDs and Gd/Ru-CDs; (**e**) XPS survey of CDs and Gd/Ru-CDs; High-resolution XPS spectra of Gd/Ru-CDs, (**f**) C1s, (**g**) N1s, (**h**) O1s, (**i**) Ru3p, and (**j**) Gd4d

Metal doping was performed via a one-step microwave-assisted synthesis procedure using BPEI, citric acid, Ru(dcbpy)_3_Cl_2_, and GdCl_3_ as precursors (Fig. 1a). In this formulation, citric acid plays a multifaceted role: serving as a carbon source, a complexing agent for the gadolinium ion, and promoting condensation with the BPEI amino group through its carboxyl component. Concurrently, BPEI functioned as a nitrogen dopant while forming complexes with Ru(dcbpy)_3_Cl_2_, engendering red fluorescence. To optimize the experimental conditions, we attempted to synthesize carbon dots with different metal doping ratios, as shown in Table S1. Gd/Ru CDs with an input mass ratio of 10:1 not only exhibits good fluorescence properties, but also has considerable potential for magnetic resonance imaging (Figures S1&S2). Therefore, it was selected for the following study. The ensuing Gd/Ru-CDs exhibited favorable water dispersion, characterized by a nearly uniform 4.2 nm diameter as evidenced by TEM imaging (Fig. 1b). Compared with undoped CDs (Figure S3), the average particle size of Gd/Ru CDs is almost close, and there is no significant change in the particle shape. High-resolution TEM analysis further discerned an individual Gd/Ru-CDs lattice spacing of 0.21 nm, aligning with graphitic structural properties (Fig. 1c) [36]. DLS assessments substantiated these findings, denoting an average hydrodynamic diameter of approximately12.4 nm, harmonizing with TEM observations (Figure S4). The zeta potentials of Gd/Ru-CDs were measured to be 20.3 mV, similar with undoped CDs (18.6 eV), attributed to the abundant presence of amino groups on their surface (Table S2). ICP-OES measurements quantified the Gd and Ru constituent at roughly 200 μg and 70 μg per mg of Gd/Ru-CDs, respectively. XRD analyses elucidated the crystalline nature of the CDs and Gd/Ru-CDs, both revealing predominant reflections around 22.1° attributable to a turbostatic layer of graphitic carbon (Fig. 1d) [37]. This, juxtaposed with a broad and somewhat noisy spectrum, hinted at the material’s underlying amorphous carbon composition.

FTIR was performed to illustrate the surface functional groups of Gd/Ru-CDs. As can be seen from Figure S5, the broad band around 3000–3500 cm^− 1^ clearly confirmed the existence of O-H and N-H bonds, which are mainly caused by its stretching vibration. Meanwhile, the peak at 2768 cm^− 1^ corresponding to -NH vibrations, indicating the presence of -NH_2_ in Gd/Ru-CDs. In addition, the existence of C = O and C=N bonds is evidenced by two peaks located at 1740 and 1350 cm^− 1^, respectively. The peak at 1180 cm^− 1^ belonged to the stretching vibration of the C-C and C-N bonds. All the above evidence shows the surface of Gd/Ru-CDs contains a variety of functional groups of hydroxyl, carboxyl and amino. Subsequent XPS evaluations mapped the chemical and elemental landscape of the Gd/Ru-CDs, ascertaining a composition rich in C, O, N, Ru, and Gd elements (Fig. 1e). In stark contrast, there are no characteristic peaks of Gd and Ru in the survey XPS spectrum of undoped CDs. Delving deeper, the C1s, N1s, and O1s spectra of both CDs and Gd/Ru-CDs demystified the presence of varied bonding environments, encompassing C = C, C-C, C-O, C-N, C-OH, and O-C-O configurations, highlighting the roles of oxygen-bearing groups and nitrogen doping agents (Fig. 1f-h&S6). Notably, the high-resolution spectrums of Gd/Ru-CDs delineated peaks pertinent to Ru 3p at 464.7 and 485.3 eV, and Gd 4d at 142.7 and 147.7 eV, corroborating the incorporation of Gd and Ru within the Gd/Ru-CDs framework (Fig. 1i-j).

**Fig. 2:**
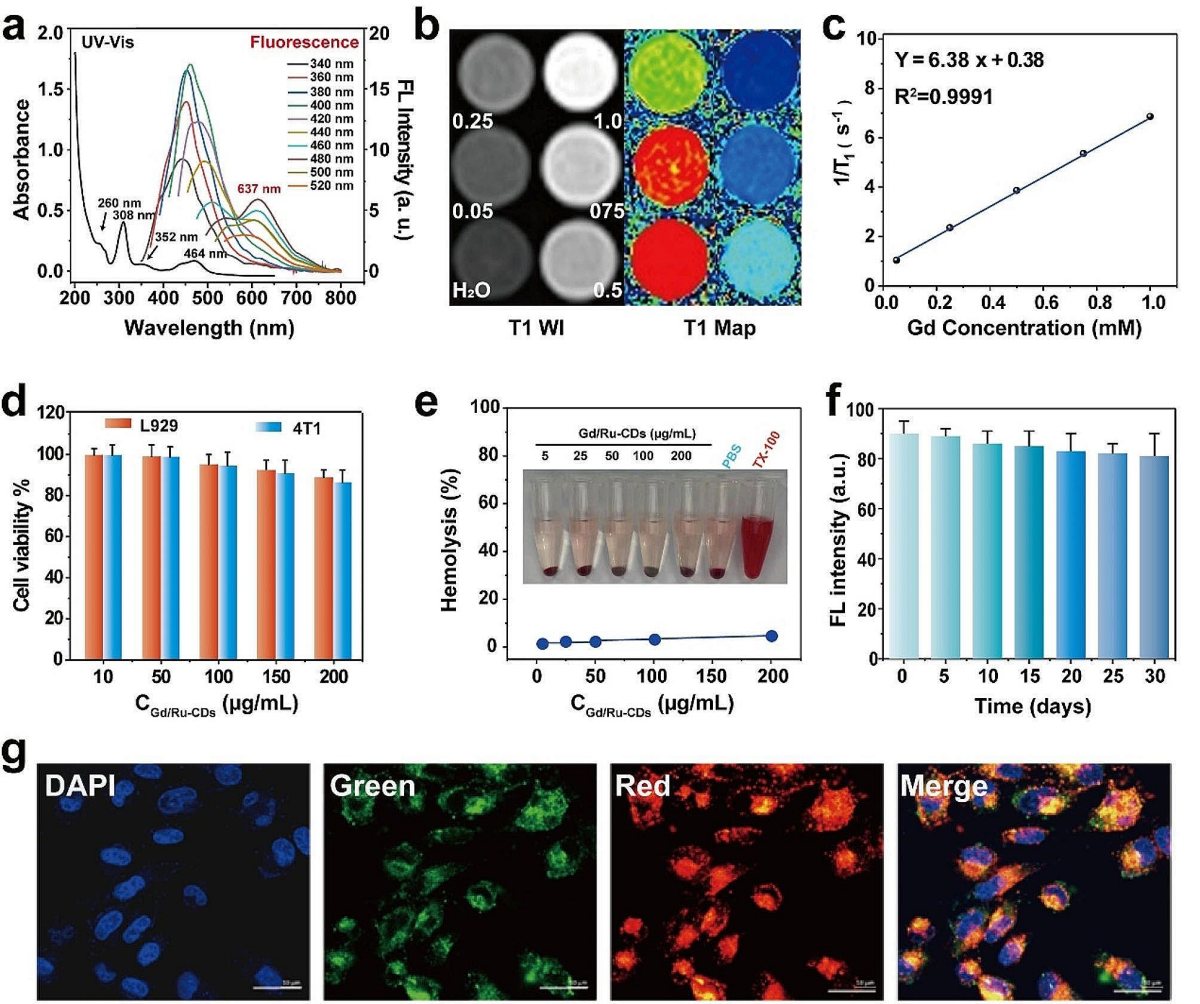
(**a**) UV-vis absorption and fluorescence spectra of Gd/Ru-CDs solutions. (**b**) T1WI and T1 Map images of Gd/Ru-CDs (Gd^3+^ concentration: mM); (**c**) and longitudinal MR relaxation curve of Gd/Ru-CDs. (**d**) Cell viability of Gd/Ru-CDs towards L929 and 4T1 cells; (**e**) The hemolytic percentage of Gd/Ru-CDs to human red blood cells (PBS as a negative control and TX-100 as a positive control); (**f**) The fluorescence stability of Gd/Ru-CDs in 30 days. (**g**) Fluorescence images of the cellular uptake of Gd/Ru-CDs (200 μg mL^− 1^) by 4 T1 cells in 6 h, scale bar = 20 μm

The optical properties of Gd/Ru-CDs were scrutinized, with UV-visible absorption spectra portraying a characteristic band at 260 nm, indicative of π-π* transitions and suggestive of the aromatic ring structures inherent to the Gd/Ru-CDs (Fig. 2a). These structures emerge through microwave-assisted carbonization, spearheaded by reactions involving diverse functional groups, such as hydroxyl, carboxyl, and amino entities present in the precursors. Noteworthy are the distinct peak at 352 nm attributable to n-π* electronic transitions facilitated by the energetic apprehension of surface oxygen functional groups in their excited states. Similarly, the UV-Vis absorption spectra of undoped CDs exhibit peaks at the same positions (Figure S7). The absorption peaks at 306 and 464 nm are consistent with that of bipyridine ruthenium dye, indicating the successful introduction of ruthenium dye. (Figure S8). Fluorescence emission spectra of both CDs and Gd/Ru-CDs, acquired under varied excitation wavelengths, exhibited a dependency on the excitation wavelength, a phenomenon possibly rooted in the heterogeneous chemical makeup and differing surface emission traps, or perhaps through mechanisms yet unidentified (Fig. 2a&S7) [38]. It is worth noting that the fluorescence spectrum of Gd/Ru-CDs exhibits a fixed red emission peak near 637 nm, and its behavior remains unchanged compared to undoped CDs, which may be attributed to the structure of Ru complexes. The utilization of longer excitation/emission wavelengths stands to benefit bioimaging applications, enhancing the penetration depth in biological tissues, a fact underscored by a recorded photoluminescence quantum yield (QY) of around 29.57% in water.

The contemplation of relaxivity in nanoprobes is central in magnetic resonance imaging quality assurance. As shown in Figure S9, compared with water, CDs without doping Gd element or only doping Ru element did not show significant T1 shortening. However, when doped with Gd elements, Gd/Ru CDs exhibit significant concentration dependent T1 changes. This indicates the excellent MRI performance of Gd doped carbon dots, and indirectly proves the successful doping of Gd elements. Subsequently, to pursue the T1 relaxation time of Gd/Ru-CDs, a series of measurements were orchestrated on a 3.0 T MR scanner, escalating the Gd concentration from 0 to 1.0 mM, and thereby establishing the vivid augmentation in the brightness of the T1-weighted MR images of the dispersions with increasing Gd concentration (Fig. 2b). Subsequently, a linear fit between 1/T1 and the Gd concentration revealed an r1 relaxation rate of 6.38 mM^-1^s^-1^, which can comparable with clinical contrast agents Gd-DTPA with r1 of 4.9 mM^-1^s^-1^, pinpointing the pivotal role of proximate interactions between the paramagnetic ions at the surface and the surrounding water molecules in dictating T1 relaxation enhancement Fig. 2c) [39]. The notably diminutive size and hydrophilicity of Gd/Ru-CDs fostered such interactions, granting them a relatively lofty r1 relaxation rate, thus heralding their aptitude for deployment as potent T1 nanoprobes in MRI applications. This showcases a prospective pathway to honing in on diagnostics with heightened precision, potentially paving the way for more insightful and accurate biomedical imaging.

Before venturing into broader biological applications, the synthesized Gd/Ru-CDs had their biocompatibility rigorously analyzed using a CCK-8 assay on 4T1 and L929 cells. A promising revelation was noted in Fig. 2d, which illustrated that both the 4T1 cells and L929 cells retained over 83% viability after a 24-hour co-incubation period, signaling a negligible cytotoxic effect of Gd/Ru-CDs. This is mainly due to the inert carbon shell that prevents Gd leakage or migration (Figure S10). Hemocompatibility was scrutinized next, through hemolysis observations conducted on red blood cells exposed to an array of substances including TX-100, PBS, and graded solutions of Gd/Ru-CDs. A comforting result emerged, evidenced in Fig. 2e, which exhibited minimal hemolysis across PBS and all Gd/Ru-CDs treated groups, mirroring the control batch. A noteworthy revelation was the nanoprobe’s superb water-dispersible trait and retention of stability, demonstrating no marked aggregation or precipitation even after a span of two months under standard conditions (Fig. 2f). These observations usher in a great signal, corroborating the high biocompatibility credential of Gd/Ru-CDs for biological deployments. Venturing into the realm of fluorescent bioimaging, cellular uptake studies in 4T1 cells unveiled a striking potential of the Gd/Ru-CDs as nanoprobes. As delineated in Fig. 2g &S11, a marked augmentation in intracellular green/red fluorescence over time (1 h, 2 h, 6 h) was visible, affirming their potent imaging efficacy in tumor cells.

**Fig. 3:**
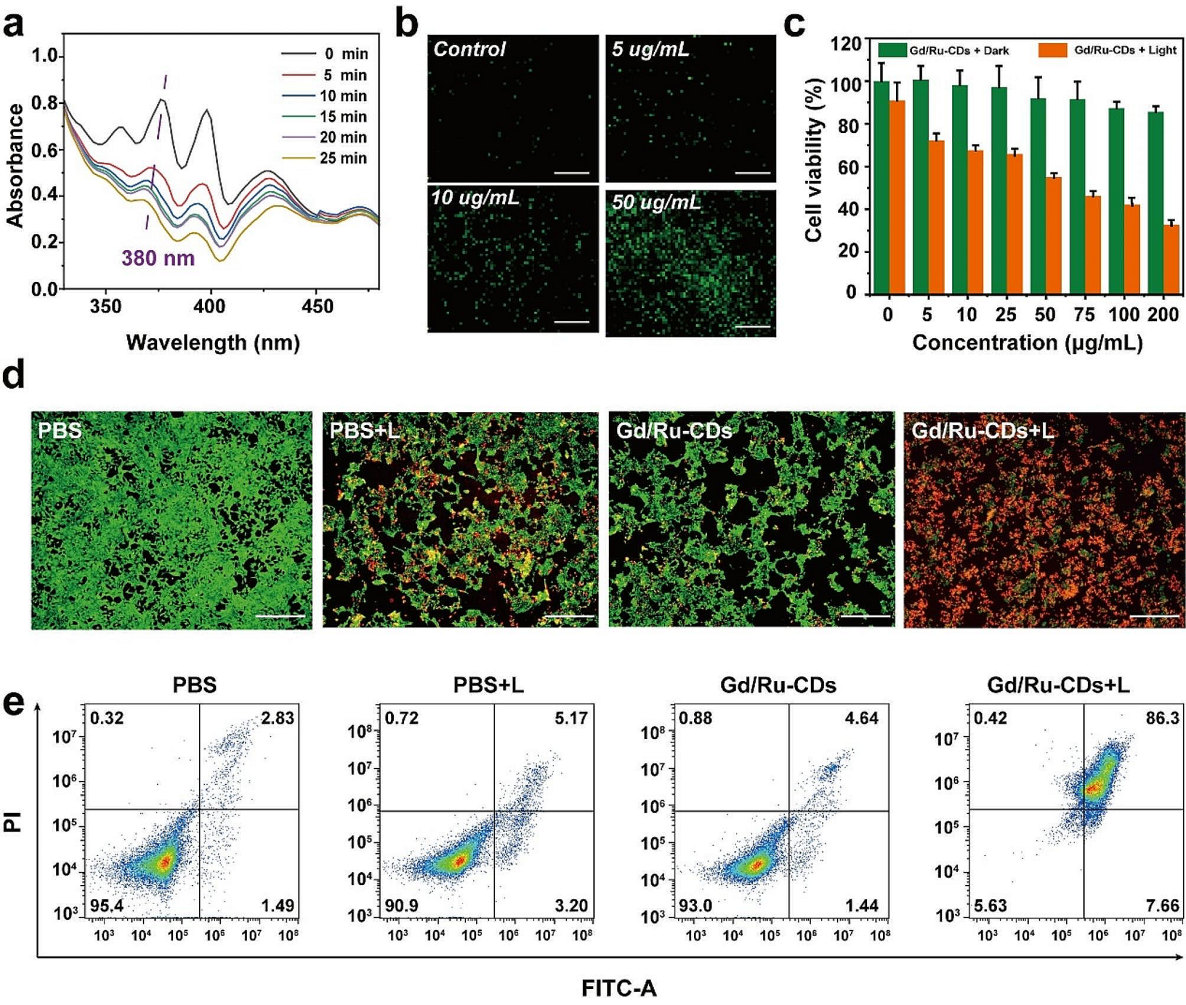
(**a**) UV-vis absorption intensity of ABDA at 380 nm after the addition of Gd/Ru-CDs and then irradiation for different time periods. Inset: the corresponding UV-vis absorption spectra. (**b**) Intracellular ROS generation of 4T1 cells incubated with Gd/Ru-CDs and probe (DCFH-DA); (**c**) Viability of 4T1 cells with and without 650 nm excitation for 10 min after incubation with different concentrations of Gd/Ru-CDs for 6 h

Photodynamic therapy (PDT), a process founded on light-induced therapeutic action, stands as a safe pathway to obliterate diseased tissues [40]. Ruthenium metal complexes have been proven to be effective PDT photosensitizers as they can absorb energy under light and release energy to transfer it to the surrounding oxygen, producing highly active singlet oxygen [41, 42]. Leveraging this concept, the Gd/Ru-CDs showcased an impressive ability to generate singlet oxygen (¹O_2_), a potent oxidizing agent critical in PDT. Through interaction with ABDA, a recognized tool for evaluating ¹O_2_ production owing to its absorbance reduction at 380 nm when reacting with ¹O_2_, the high efficiency of ¹O_2_ generation from Gd/Ru-CDs was corroborated. As shown in Fig. 3a, a remarkable 80% absorption decay observed within a span of 20 min post-irradiation using an LED lamp substantiated this efficiency. The study proceeded with assessing the reactive oxygen species (ROS) production within 4T1 cells utilizing the DCFH-DA probe, revealing a discernible green fluorescence only upon Gd/Ru-CDs exposure and subsequent white light irradiation, confirming the ROS induction capability solely in the presence of light and Gd/Ru-CDs (Fig. 3b). When scrutinizing the PDT efficiency, a drastic decline in the viability of 4T1 cells, spotlighted in Fig. 3c, was evidenced post a 10-minute irradiation session at 650 nm wavelength, underscoring a notable PDT efficacy while retaining a low toxicity profile.

The in vitro photodynamic therapeutic effect was firstly evaluated on 4 T1 tumor cells by the live/dead cell staining assay. 4T1 cells were incubated with (i) Saline, (ii) Saline + L, (iii) Gd/Ru-CDs, (v) Gd/Ru-CDs + L and co-stained with calceinAM and PI. As depicted in Fig. 3d, **4T1** cells in the control (Saline) revealed vivid green fluorescence, indicating the good living condition of the cells. Moreover, cells in saline + L, Gd/Ru-CDs groups also exhibited strong green fluorescence, demonstrating that they could not induce any strong injury to the tumor cells. However, when combined with laser irradiation, Gd/Ru-CDs efficiently brought about most of cell death, which were stained to be red in the field. Next, an Annexin V-FITC/PI fluorescence staining was also performed on the cells and analyzed by a flow cytometry system. As shown in Fig. 3e, more than 90% of cells were found to be distributed in the left lower quadrant for the saline, saline + L, Gd/Ru-CDs without laser irradiation group, indicating the good living condition of the cells after the above treatments. However, after laser irradiation, the apoptosis rate achieved 94.4% for the Gd/Ru-CDs incubated cells, respectively. The flow cytometry data suggested that cells treated by various Gd/Ru-CDs displayed irreversible damage upon photodynamic treatment. The photodynamic antitumor effect of the various groups was further quantitatively assessed by the CCK-8 assay. As shown in Figure S12, without laser irradiation, Gd/Ru-CDs did not induced evident damage to the 4T1 cells compared to the control group. However, in contrast, the cell viabilities decreased to about 58% for the Gd/Ru-CDs exposed to 650 nm laser, respectively, attributed to their photodynamic effect. Thus, these results indicated that the Gd/Ru-CDs would be a great potential PDT application in cancer treatment.

**Fig. 4:**
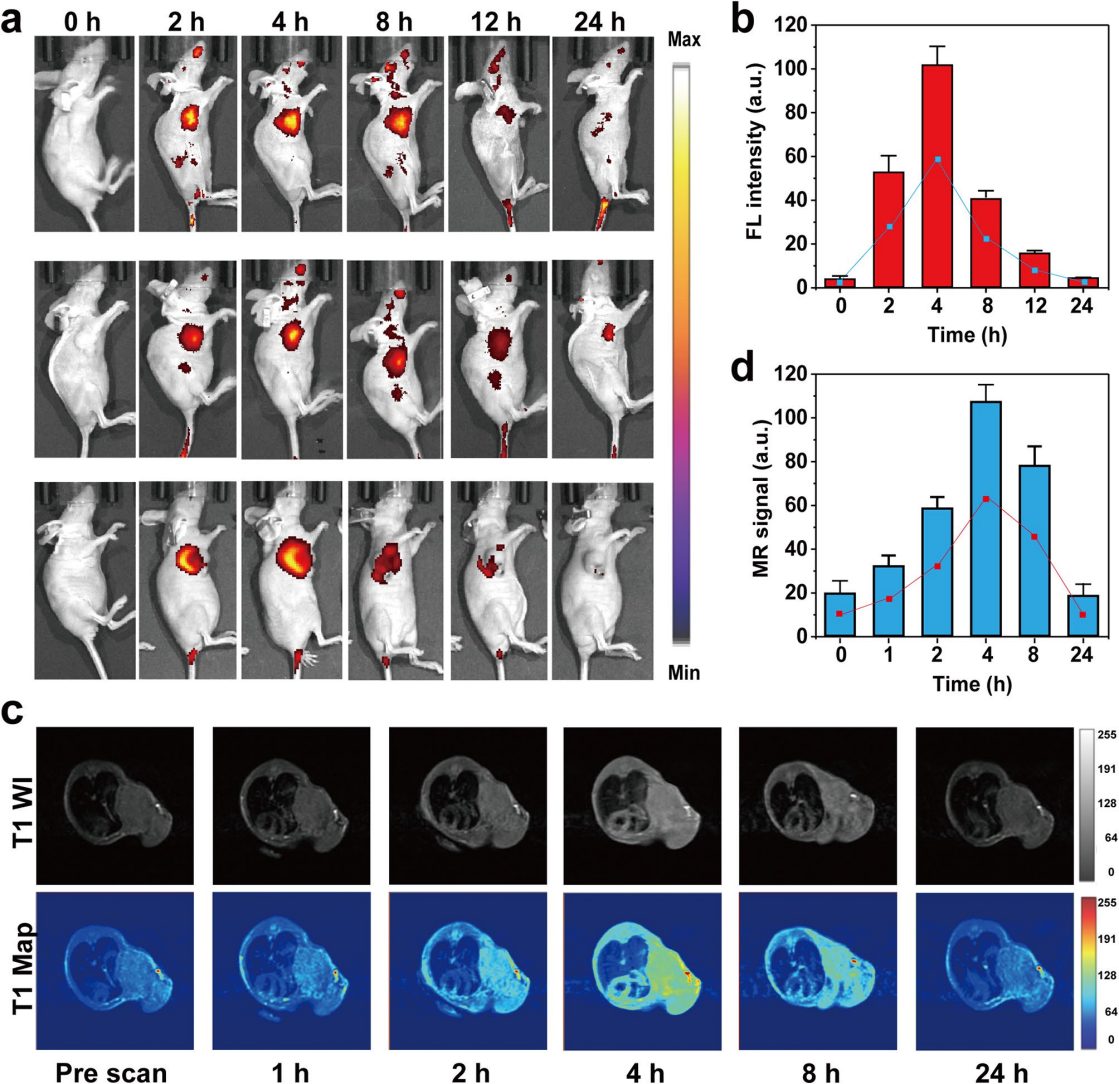
In vivo fluorescence and MR imaging (three mice per group). (**a**) In vivo fluorescence imaging of 4T1 tumor-bearing mice after intravenous injection of Gd/Ru-CDs; (**b**) FL signal intensities within tumor regions at corresponding time points after intravenous injection of Gd/Ru-CDs; (**c**) T1-weighted MR imaging of mice model pre- and post-injection of Gd/Ru-CDs at various time intervals; (**d**) T1-weighted MRI signals of the tumors at determined time points after administration of Gd/Ru-CDs

An empirical venture was undertaken to demonstrate the tumor imaging and imaging-guided therapy capabilities of Gd/Ru-CDs in a 4T1 xenograft mouse model. Due to enhanced permeability and retention (EPR) effects, prominent fluorescent signals of Gd/Ru-CDs accumulation in tumor tissues were witnessed 3 h post-injection, as illustrated in Fig. 4a. Tumors are easily distinguishable from surrounding tissues, with fluorescence intensity maintained for 12 h (Fig. 4b). Moreover, we use a fluorescence imaging system to measure its biological distribution in major organs and tumors. After the injection of Gd/Ru-CDs in 1 h, strong fluorescence signals were found in the bladder, indicating that the Gd/Ru-CDs may have been cleared through the kidneys (Figure S13). Dissect the mice 4 h after injection, respectively. As expected, strong fluorescence signals appeared in the tumor area. More importantly, a large amount of Gd/Ru-CDs remained in the kidneys, which further confirms that Gd/Ru-CDs can be cleared through the renal pathway (Figure S14). Subsequently, we collected urine samples after 4 h and observed strong fluorescence characteristics of Gd/Ru-CDs (Figure S15). This result was also confirmed by TEM, confirming renal clearance and high stability of carbon dot metabolism. In addition, most of the injected carbon dots were detected in urine and their content in feces was negligible, indicating that the clearance of Gd/Ru-CDs is mainly carried out through the kidneys (Figure S16). The above results indicate that Gd/Ru-CDs can effectively accumulate at the tumor site and be excreted from the body through the kidneys.

T1-weighted MR imaging conducted serially at distinct intervals pre and post intravenous Gd/Ru-CDs administration depicted a peak in tumor T1 signal intensity at the 3-hour mark, post which it gradually declined, normalizing in a 12-hour window (Fig. 4c). The nanoparticles facilitated crisp delineation of tumor peripheries, enhancing edge imaging and offering a precise demarcation between normal and tumor tissues. A commendable attribute was the prolonged retention of a high T1 signal in tumors, upheld till 5 h subsequent to the administration, showcasing potential for long-term tracking (Fig. 4d). The enhanced visualization of the tumor border in the critical 1–5 h post-injection timeframe underscored a substantial window for effective MR imaging, thereby promising stellar capabilities in tumor diagnostic imaging. This pathway illustrates not just the promising potential of Gd/Ru-CDs in crafting detailed fluorescence and MR images but potentially revolutionizes tumor diagnostics, affording a larger temporal window to carry out precise and informative MRI sessions, thereby possibly steering towards better prognostic outcomes.

**Fig. 5:**
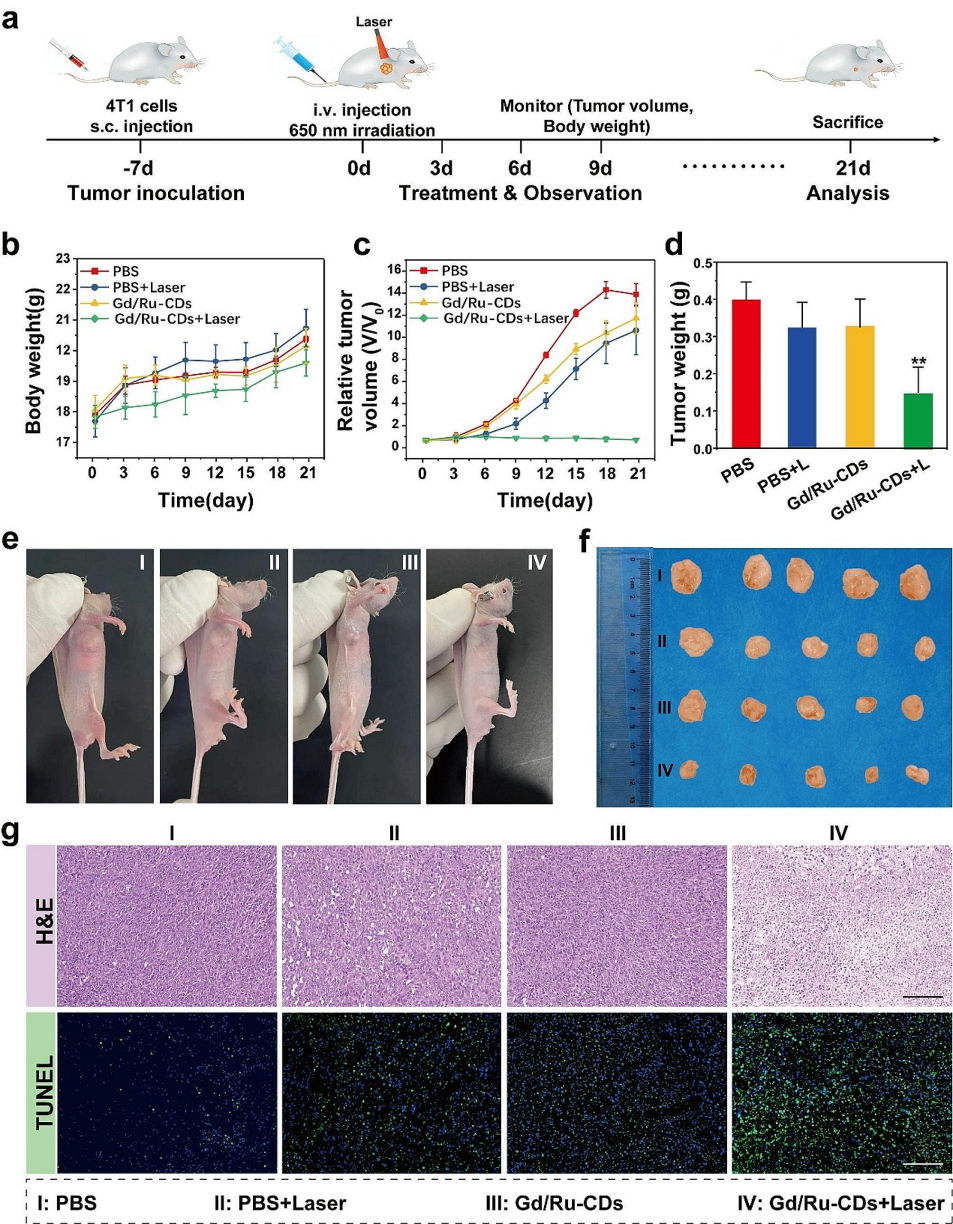
In vivo anticancer effect in 4T1 tumor bearing mice. (**a**) Schematic illustration of animal experiments design; (**b**) body weight (**c**) Relative tumor size, (**d**) Tumor weight, and (**e**) Photographs of 4T1 tumor bearing mice and the images of the tumor at 21 days for various groups: PBS, Laser only, Gd/Ru-CDs, Gd/Ru-CDs + Laser. (**h**) H&E stained and Tunnel images of the tumors from different groups. Scale bars: 100 μm

In vivo experiments evaluated the imaging-guided photodynamic therapy (PDT) efficacy of Gd/Ru-CDs in 4T1 tumor-bearing mice. This study divided mice into four different groups. The first group served as the control group and received intravenous injection of PBS solution. The second group exposed the tumor site to 650 nm laser for 20 min. Both the third and fourth groups were intravenously injected with Gd/Ru-CDs. One hour after injection, the fourth group received 20 min of 650 nm laser irradiation at the tumor site. This cycle of Gd/Ru-CDs injection followed by light exposure was repeated every three days for 21 days (Fig. 5a). A consistent observation was that all mouse groups maintained normal body weight, indicating the low toxicity of Gd/Ru-CDs and photoirradiation therapy (Fig. 5b). Fluctuations in tumor volume were carefully monitored during this period (Fig. 5c). It can be seen from the size of the mice in each group after treatment, the weight of the resected tumors and the photo records that the fourth group showed the most significant tumor suppression effect, leaving minimal tumor residues at the end of three weeks, or even completely tumor elimination (Fig. 5d-f). After the two-week experimental period, all tumors were carefully dissected to facilitate H&E and TUNEL analysis. As shown in Fig. 5g, the PDT group (Gd/Ru-CDs + laser) had severe histological damage and typical pathological changes, such as severe pyknosis, apoptosis or necrosis of tumor cells. In contrast, there was almost no tumor destruction and necrosis in the control group and other treatment groups, and the treatment effect was limited. Therefore, the emergence of Gd/Ru-CDs depicts a robust, efficient, and safe approach to cancer treatment, which is expected to revolutionize the field of PDT and herald the future of precise, efficient, and targeted cancer treatment with minimized side effects.

**Fig. 6:**
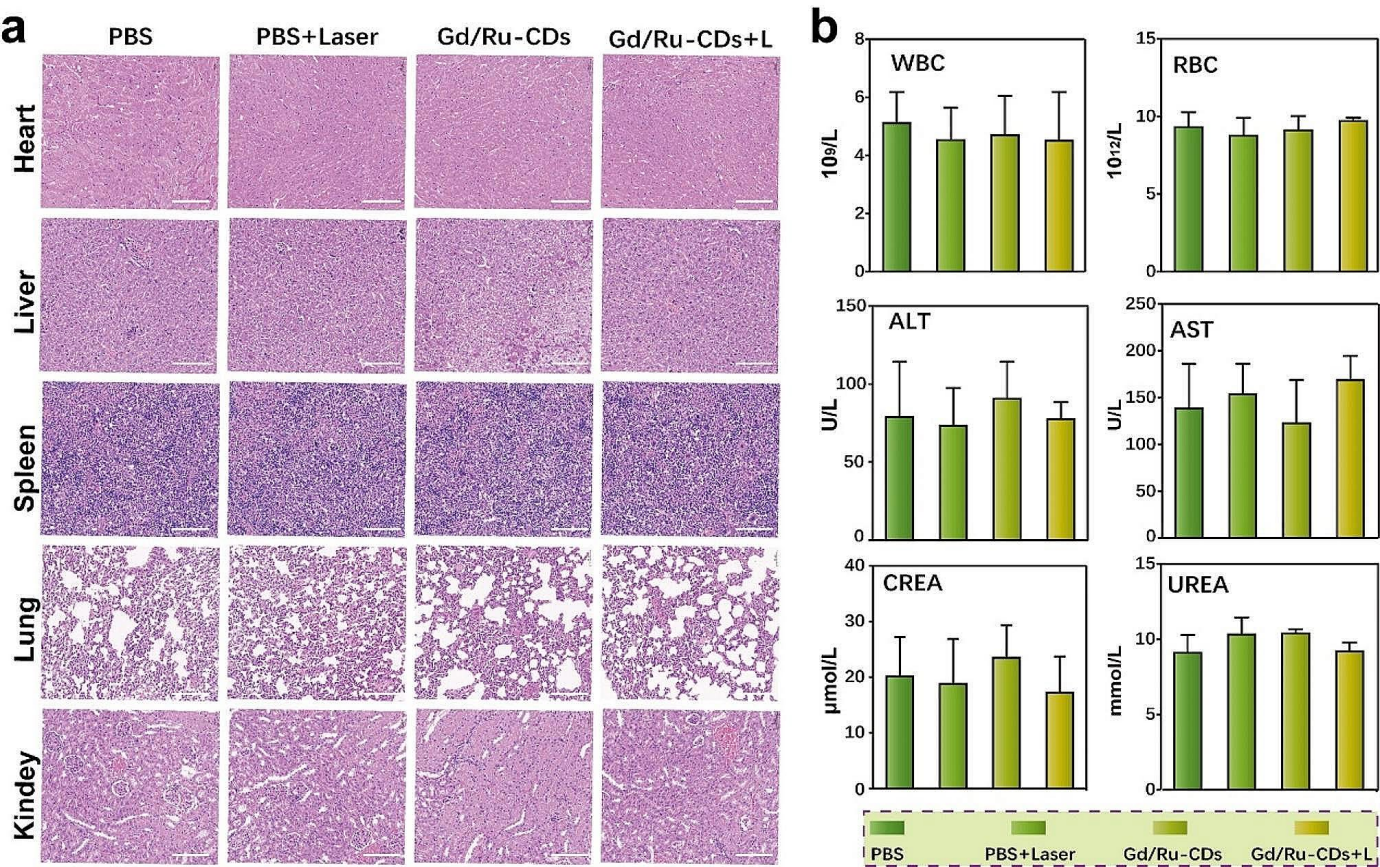
In vivo biosafety evaluation of Gd/Ru-CDs. (**a**) H&E staining of the tissue sections (Heart, Liver, Spleen, Lung, Kindey) of mice. The scale bar is 100 μm. (**b**) Detection of blood routine and liver and kidney function in mouse serum

The biocompatibility of Gd/Ru-CDs is pivotal for their potential transition into clinical settings. Therefore, to assess the in vivo biocompatibility and prospective toxicity of Gd/Ru-CDs, both serum biochemical and histological analyses were undertaken. As depicted in Fig. 6a, H&E-stained tissue sections from various mouse organs (namely the heart, liver, spleen, lung, and kidney) exhibited no histopathological abnormalities or lesions one day post Gd/Ru-CDs injection. Moreover, blood routine white blood cell (WBC), red blood cell (RBC), liver function markers, specifically alanine aminotransferase (ALT), aspartate aminotransferase (AST), along with crucial renal indicators such as creatinine (CREA) and urea (UREA) remained within the standard range across all treatment groups throughout the one-week evaluation period (Fig. 6b). These findings substantiate that Gd/Ru-CDs do not induce sustained impairment to normal renal and hepatic functions. Preliminary data thus denote the low toxicity and commendable biocompatibility of Gd/Ru-CDs nanoprobes in vivo, fostering their prospective applications in the biomedical domain.

## Conclusions

A carbon dot (Gd/Ru-CDs) based nanoprobe has been formulated using a simple one pot microwave-assisted synthesis method. This nanoprobe has excellent red emission and significant longitudinal relaxation rate (r1), and in addition to this plant, it also has low cytotoxicity and biocompatibility. The proposed Gd/Ru-CDs nanoprobes can not only achieve specific optical imaging and MR tracking of tumor cells, but also generate ROS accumulation at the tumor site for photodynamic therapy. Importantly, in addition to good biocompatibility, the probe can also be effectively expelled from the body after imaging, avoiding probe accumulation and potential long-term toxicity in the body. Thus, the devised Gd/Ru-CDs nanoprobes have shown great potential in precise imaging and guided tumor treatment.

### Electronic supplementary material

Below is the link to the electronic supplementary material.

Supplementary Material 1

## Data Availability

The data are available from the corresponding author upon reasonable request.
