# Supplementary material for: Facile synthesis of Gd/Ru-doped fluorescent carbon dots for fluorescent/MR bimodal imaging and tumor therapy
Source: J Nanobiotechnology. 2024 Mar 2;22:88. doi: 10.1186/s12951-024-02360-4 (PMC10908135; doi:10.1186/s12951-024-02360-4)
Supplement: Supplementary file 1 — Supplementary Material 1 [file 12951_2024_2360_MOESM1_ESM.docx]

**Facile synthesis of Gd/Ru-doped fluorescent carbon dots for fluorescent/MR bimodal imaging and tumor therapy**

*Yupeng Shi*, Yaning Xia, Mengyang Zhou, Yifei Wang, Jianfeng Bao, Yong Zhang, Jingliang Cheng*

Department of MRI, The First Affiliated Hospital of Zhengzhou University; Henan Key laboratory of Functional Magnetic Resonance Imaging and Molecular Imaging, Zhengzhou 450052, China；

* Corresponding Author.

Email address: [shiyup@zzu.edu.cn](mailto:shiyup@zzu.edu.cn); Yupeng Shi

**Table S1.** The proportion of raw material input for preparing carbon dots.

|  | PEI | Citric acid | Ru(dcbpy)_3_Cl_2_ | GdCl_3_ | H_2_O |
| --- | --- | --- | --- | --- | --- |
| Ⅰ | 1 g | 0.5 g | 0 | 0 | 10 mL |
| Ⅱ | 1 g | 0.5 g | 0 | 50mg | 10 mL |
| Ⅲ | 1 g | 0.5 g | 5 mg | 50 mg | 10 mL |
| Ⅳ | 1 g | 0.5 g | 10 mg | 25 mg | 10 mL |
| Ⅴ | 1 g | 0.5 g | 5 mg | 0 | 10 mL |


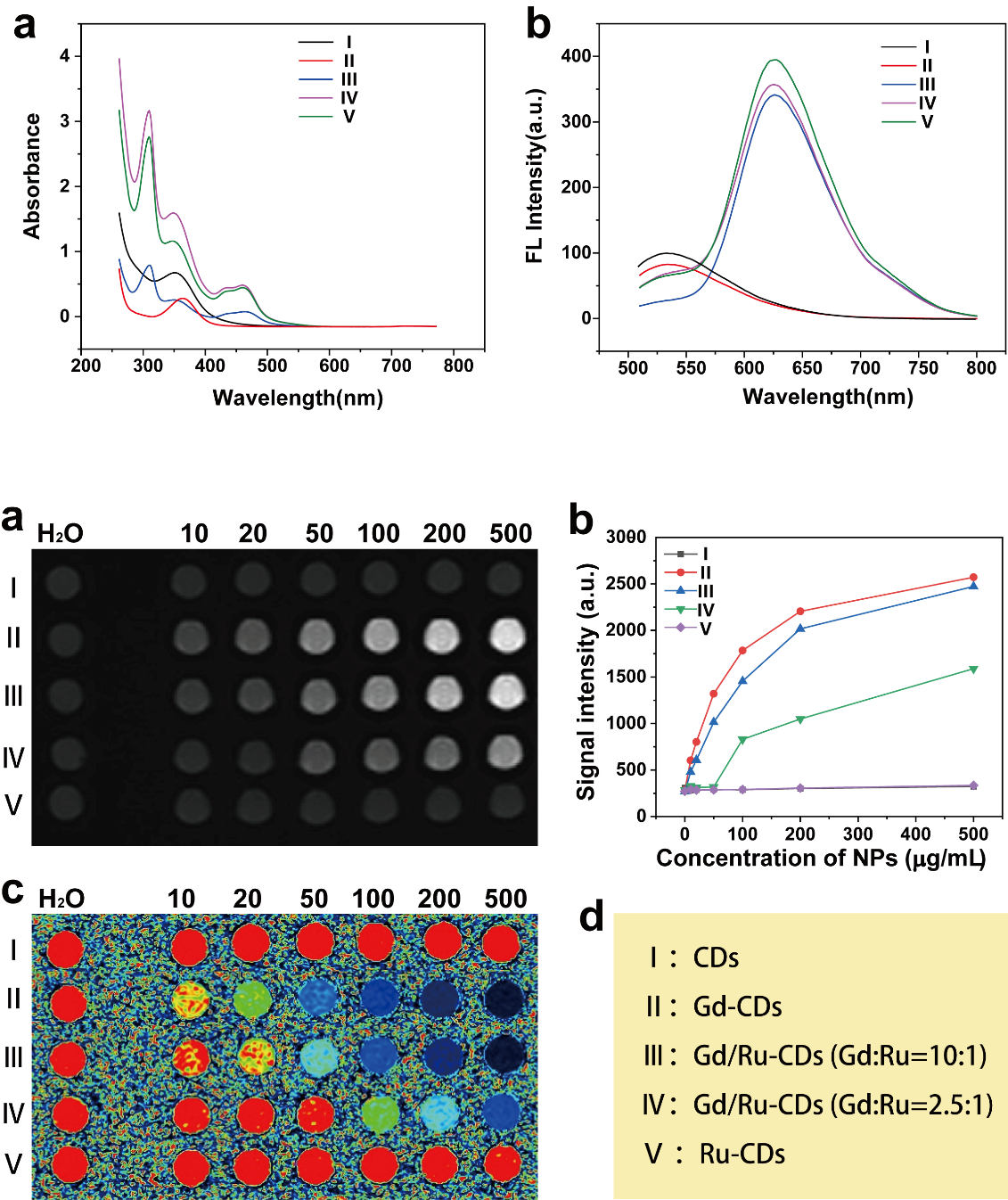


**Figure S1.** (a) UV-Vis and (b) Fluorescence spectra of the carbon dots with different synthesis conditions.


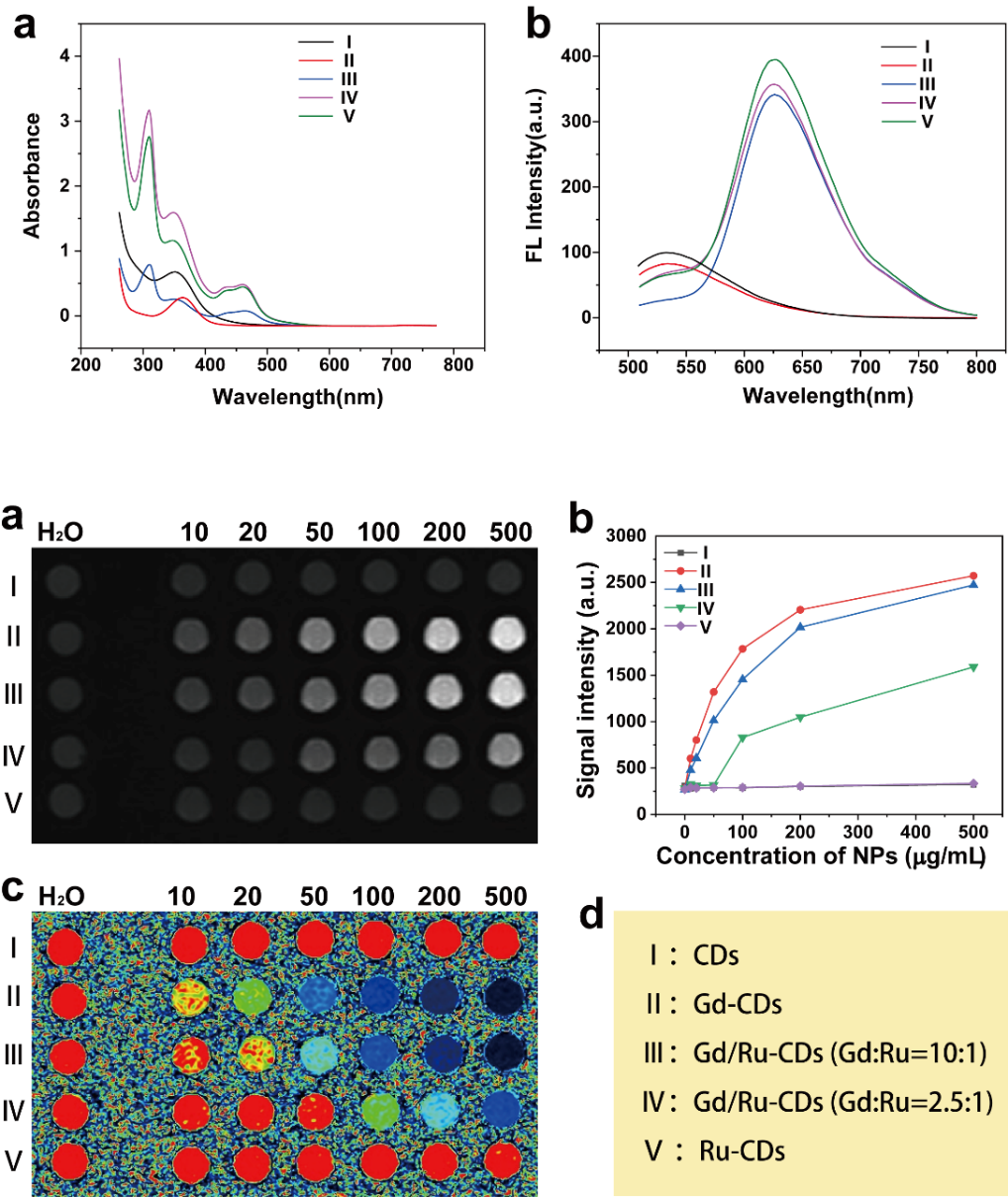


**Figure S2.** MRI properties of carbon dots with different synthesis conditions. (a) T1WI images. (b) The related signal intensity of T1WI images. (c) T1Map images. (d) The different synthesis conditions.


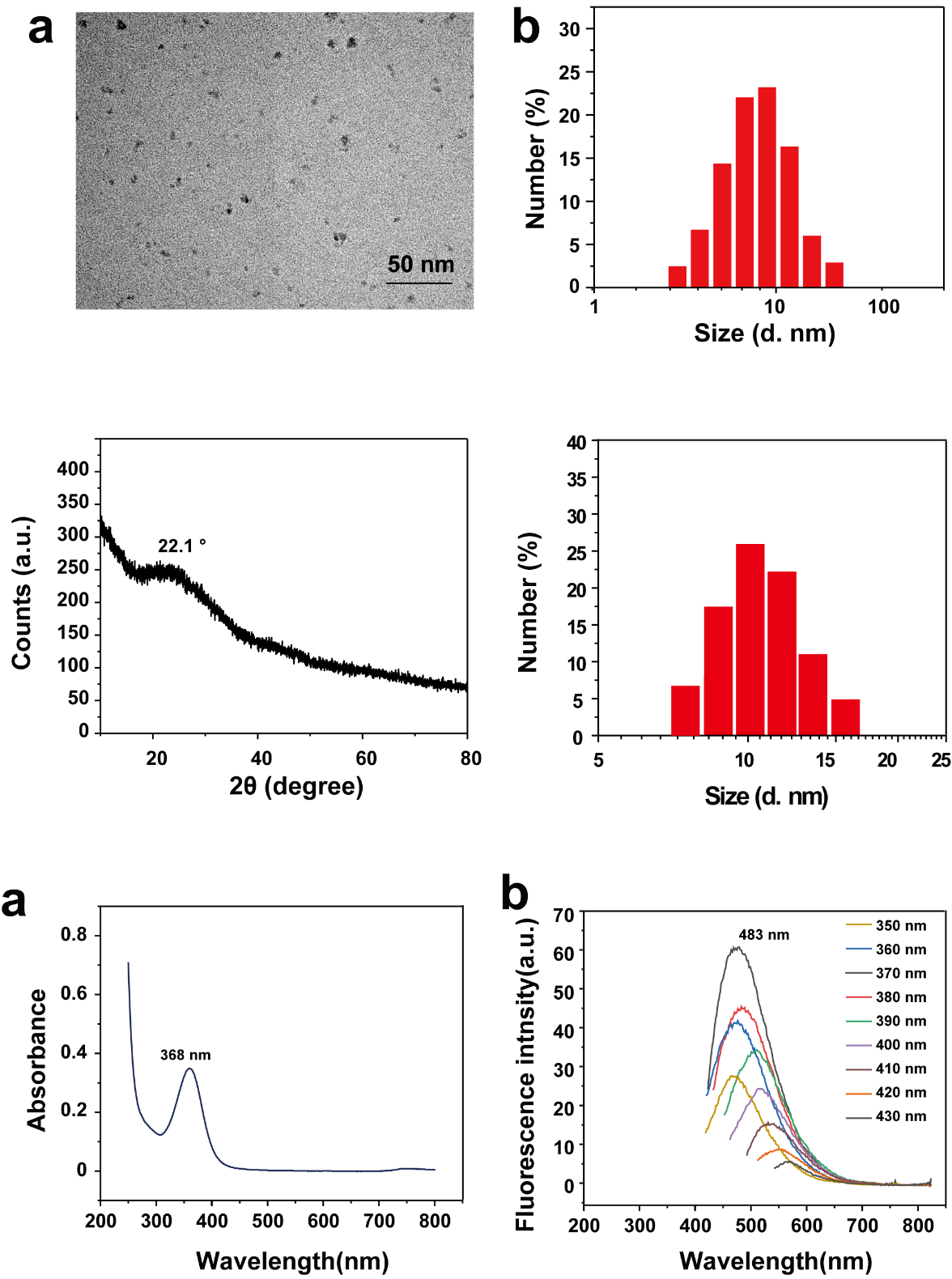


**Figure S3.** (a) TEM and (b) DLS size distribution of un-doped carbon dots (CDs).


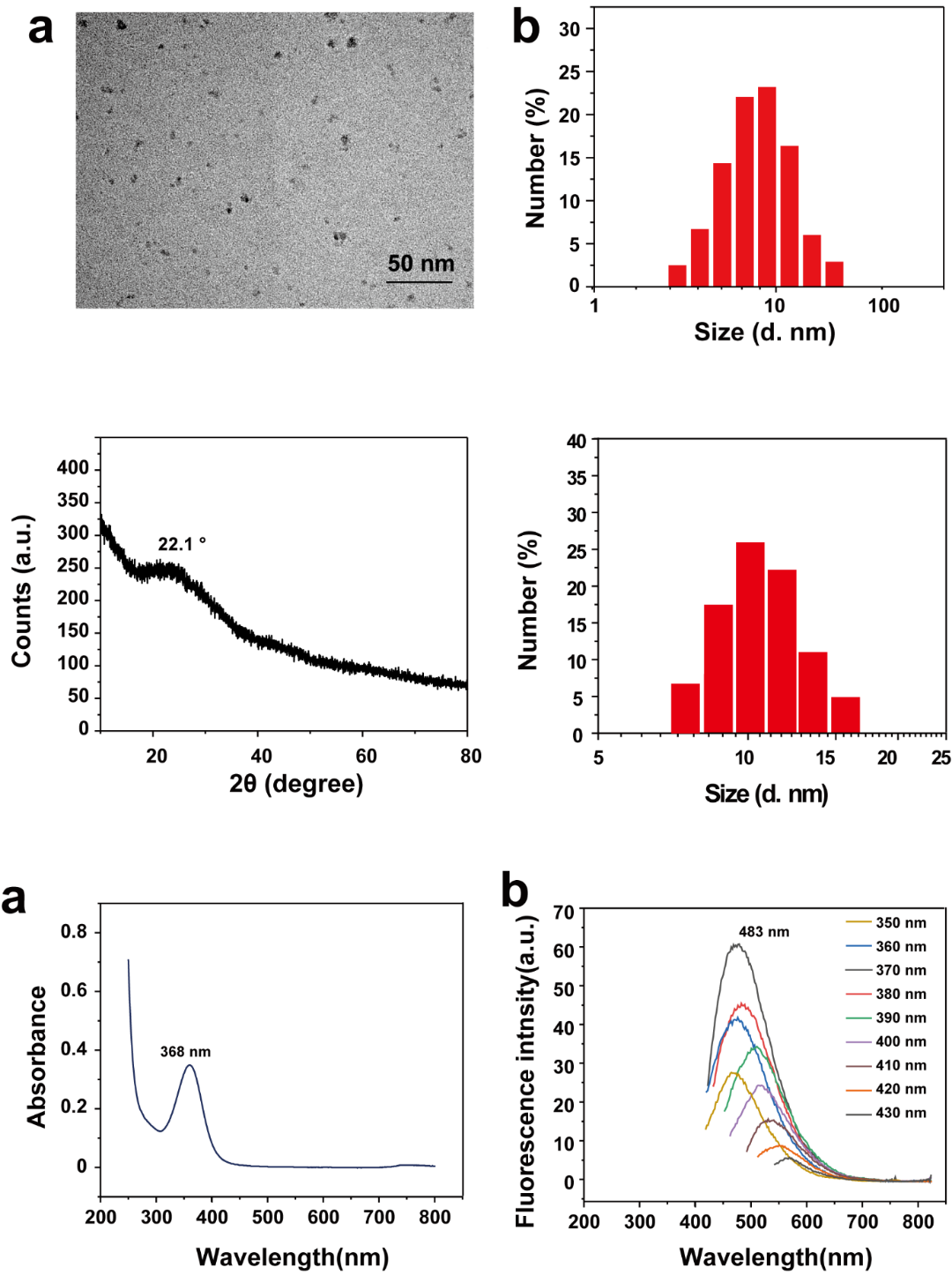


**Figure S4.** The size distribution of Gd/Ru-CDs measured by DLS.

**Table S2**. The size and zeta of CDs and Gd/Ru-CDs.

| Materials | Size (nm) | Zeta (mv) |
| --- | --- | --- |
| CDs | **16.4**±**4** | **18.6** |
| Gd/Ru-CDs | **12.4**±**3** | **20.3** |


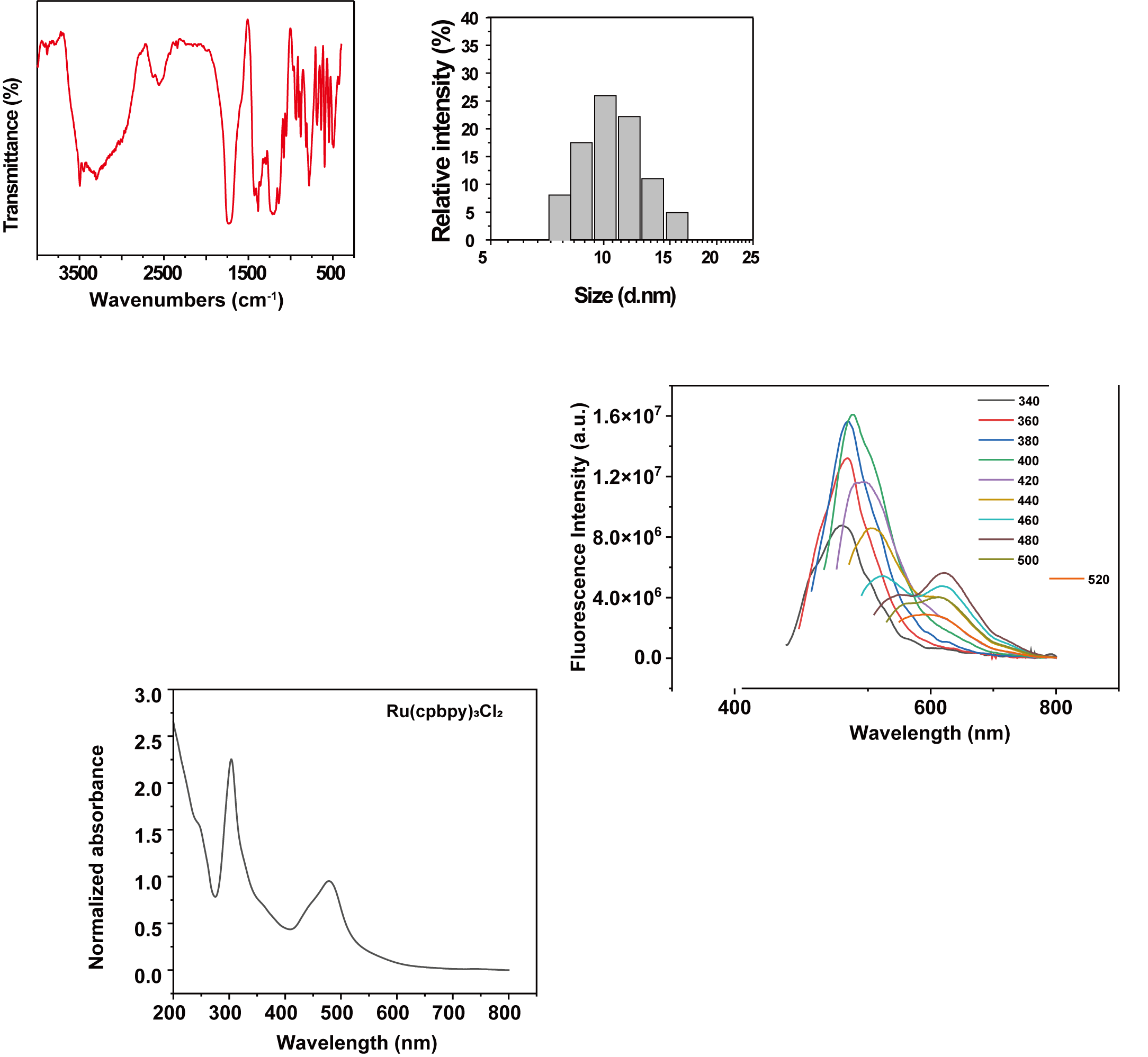


**Figure S5.** FTIR spectra of Gd/Ru-CDs.


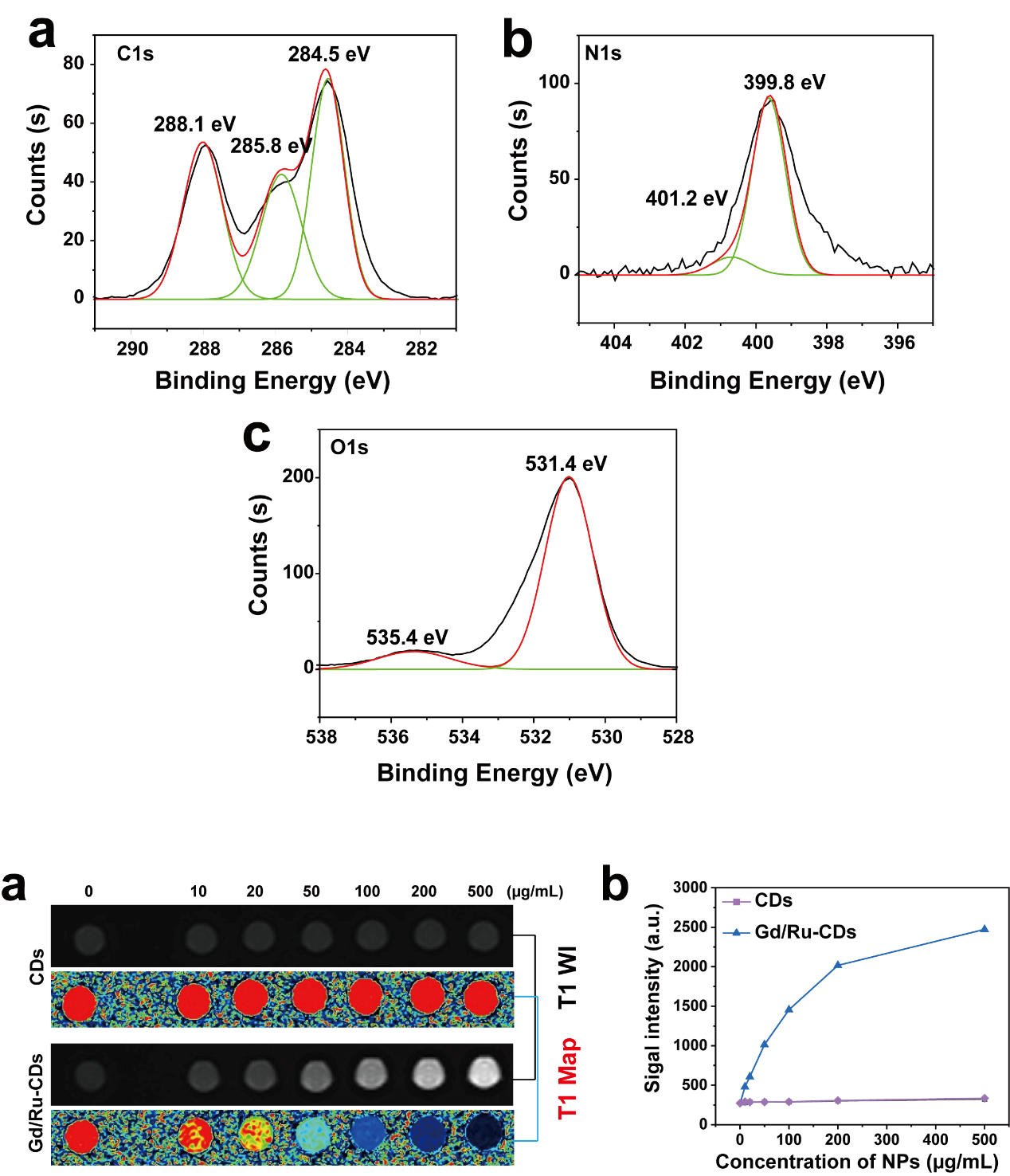


**Figure S6.** XPS spectrum of CDs. (a) C1s; (b) N1s; (c) O1s.


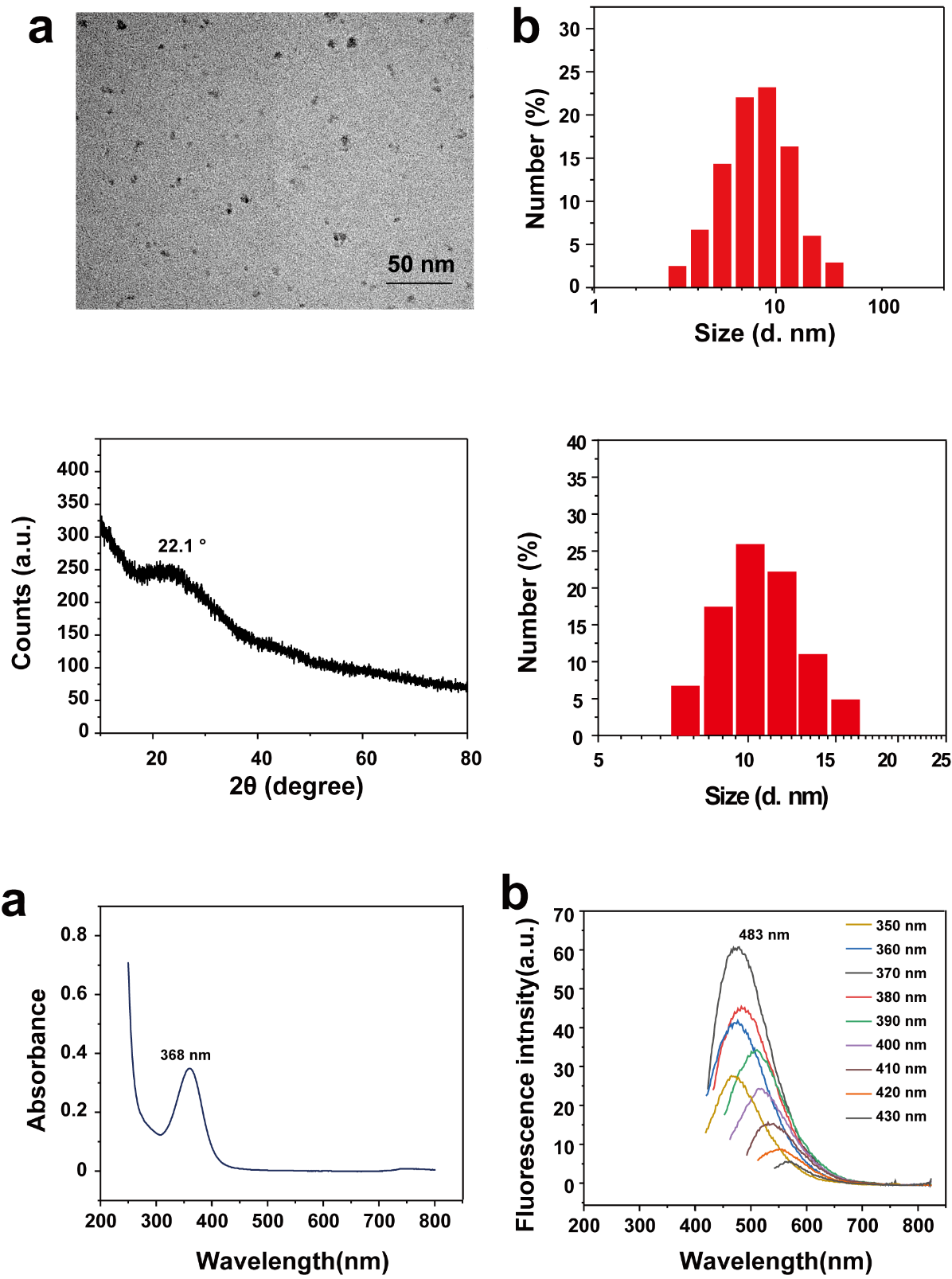


**Figure S7.** (a) UV-Vis and (b) Fluorescence spectra of CDs.


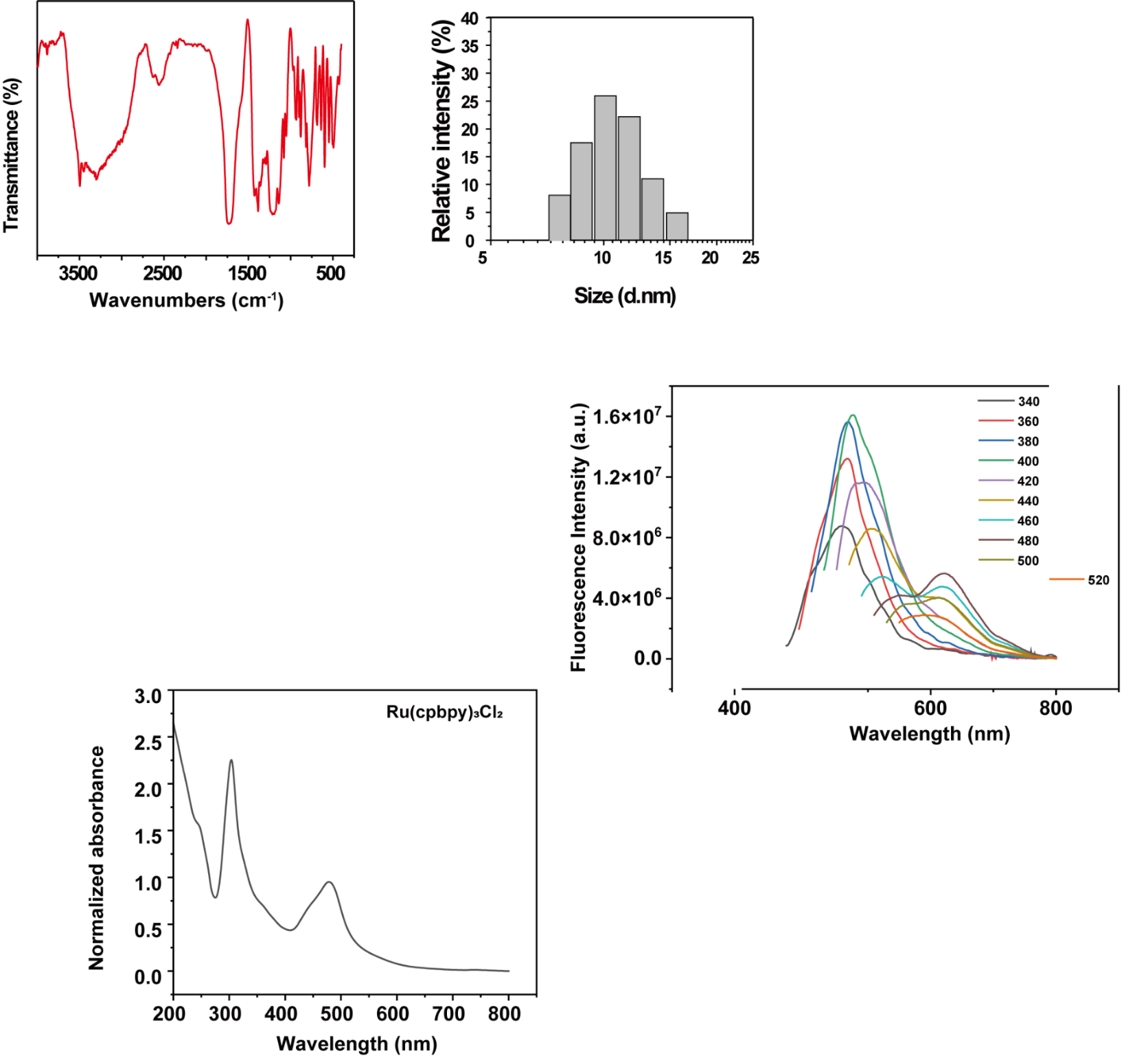


**Figure S8.** The UV-Vis spectrum of Ru(dcbpy)_3_Cl_2_.


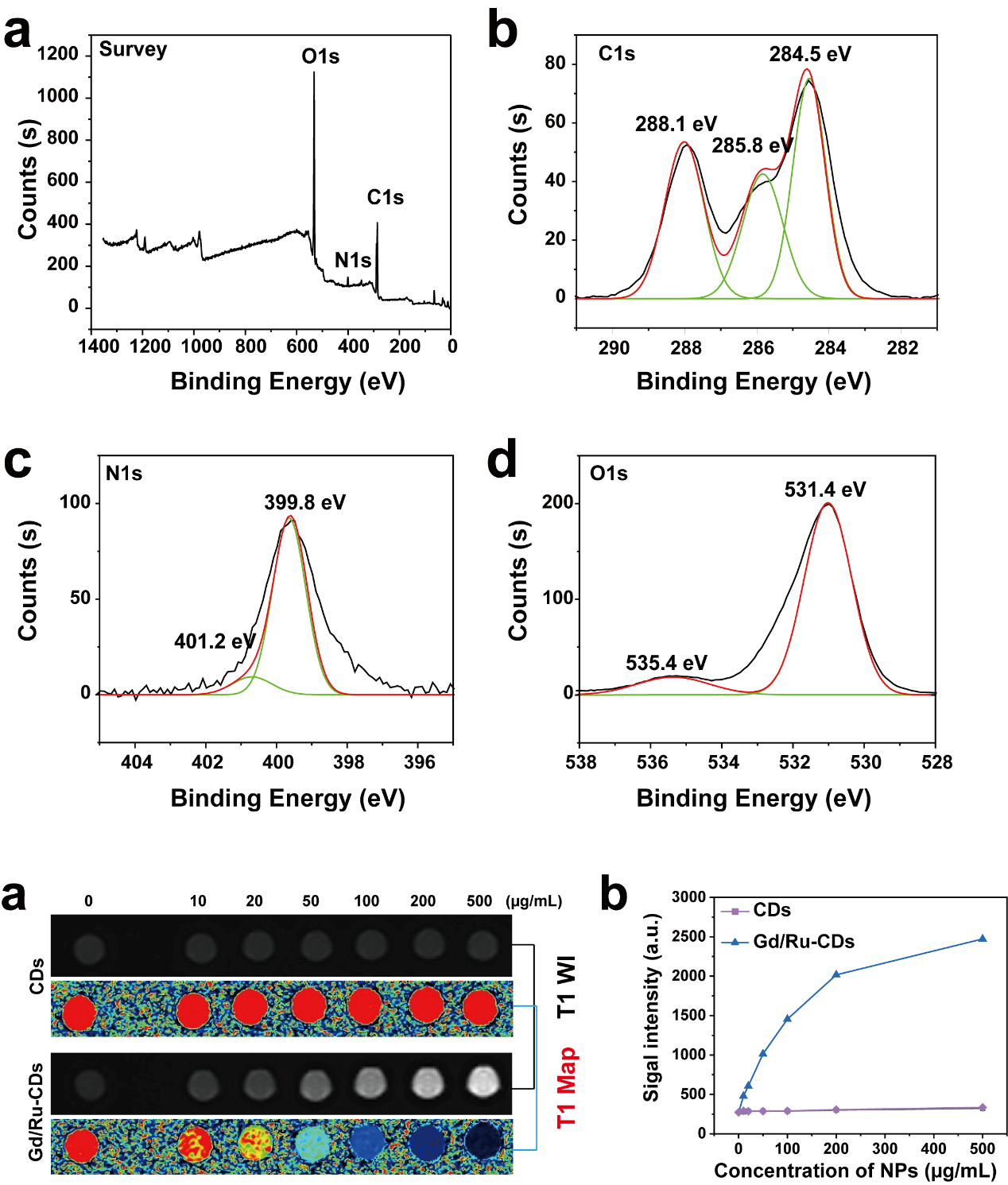


**Figure S9.** (a) MRI images with different concentrations of CDs and Gd/Ru-CDs; (b) Signal intensity of CDs and Gd/Ru-CDs with T1WI images.


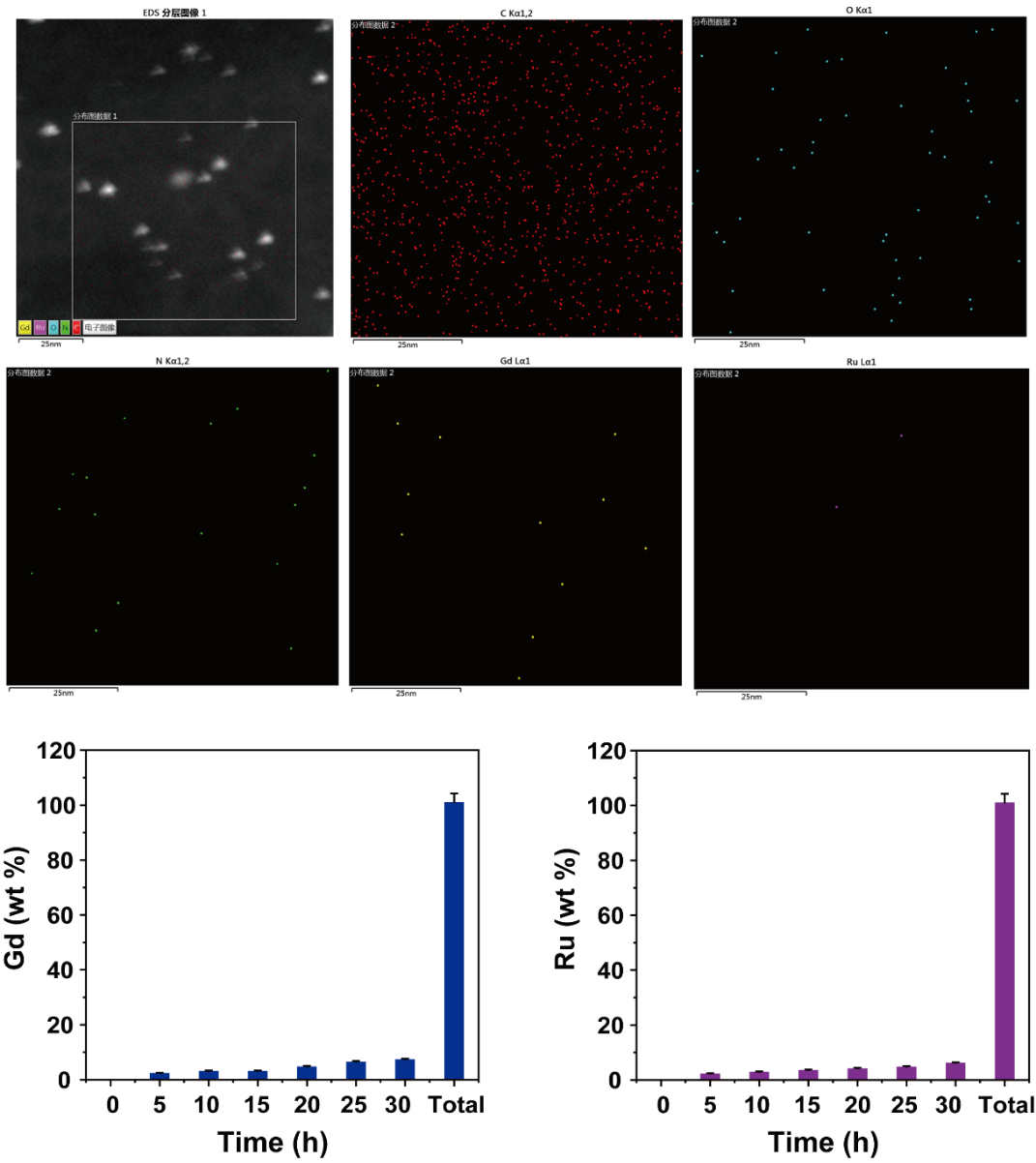


**Figure S10.** Gd and Ru leakage from the Gd/Ru-CDs in cubated in an aqueous solution. Total is the total original amount of Gd or Ru.


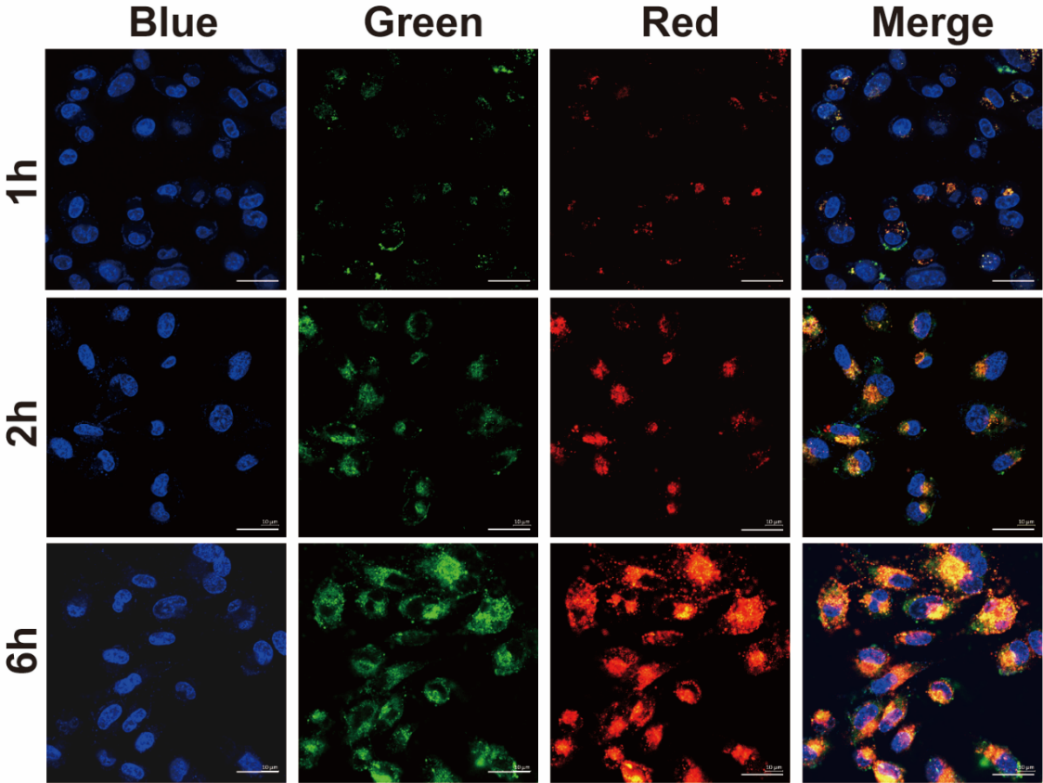


**Figure S11.** Fluorescence images of the cellular uptake of Gd/Ru-CDs (200 μg mL^-1^) by 4 T1 cells in 1 h and 2h, scale bar = 20 μm.


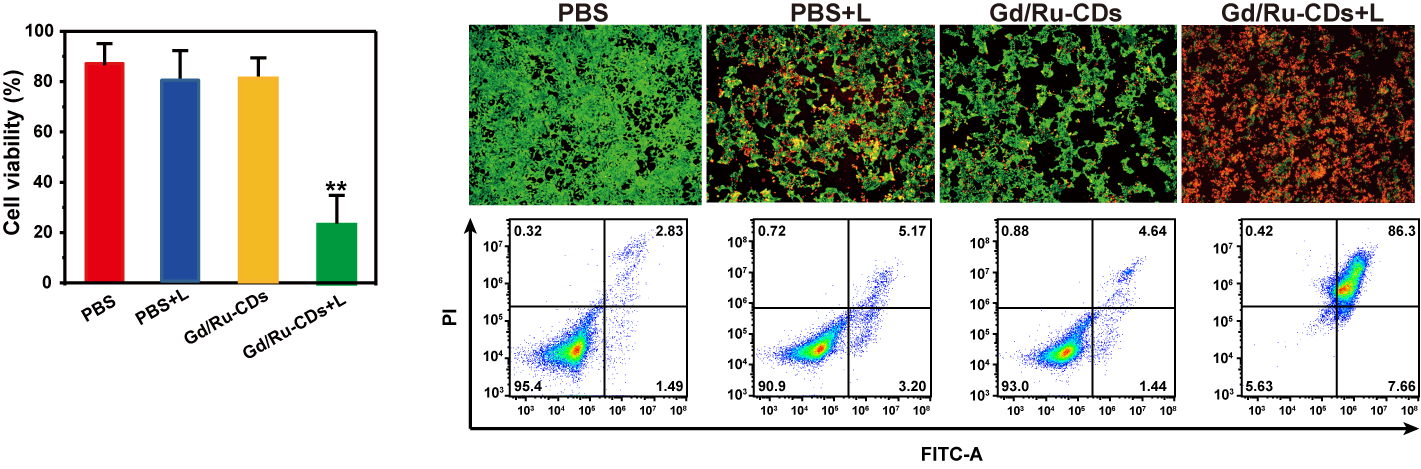


**Figure S12.** Viability of 4T1 cells after deal with different conditions (PBS, PBS+L, Gd/Ru-CDs and Gd/Ru-CDs+L).


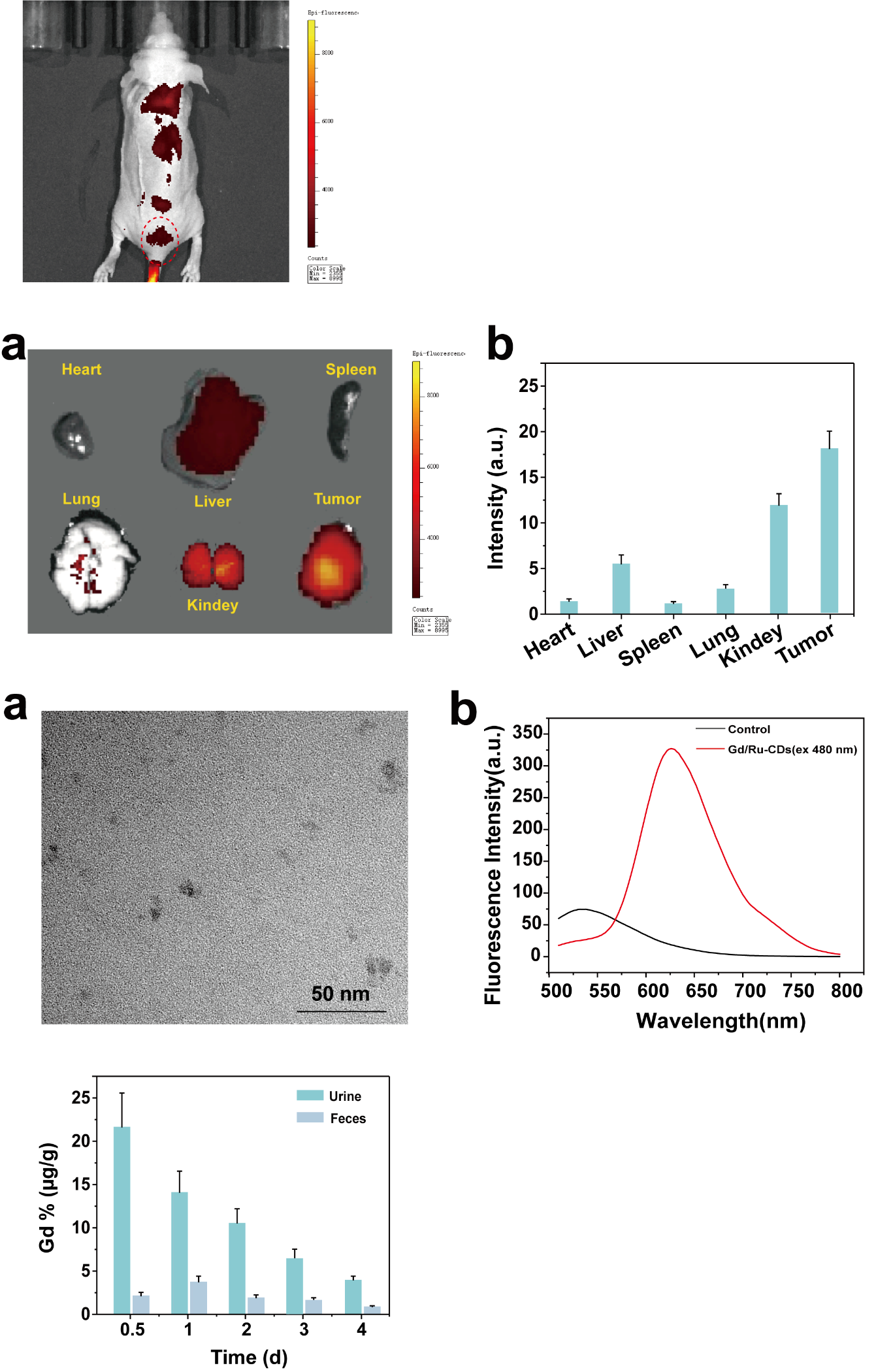


**Figure S13.** *In vivo* fluorescence image of the mice after i.v. injection of Gd/Ru-CDs at 1h (ex: 480 nm).


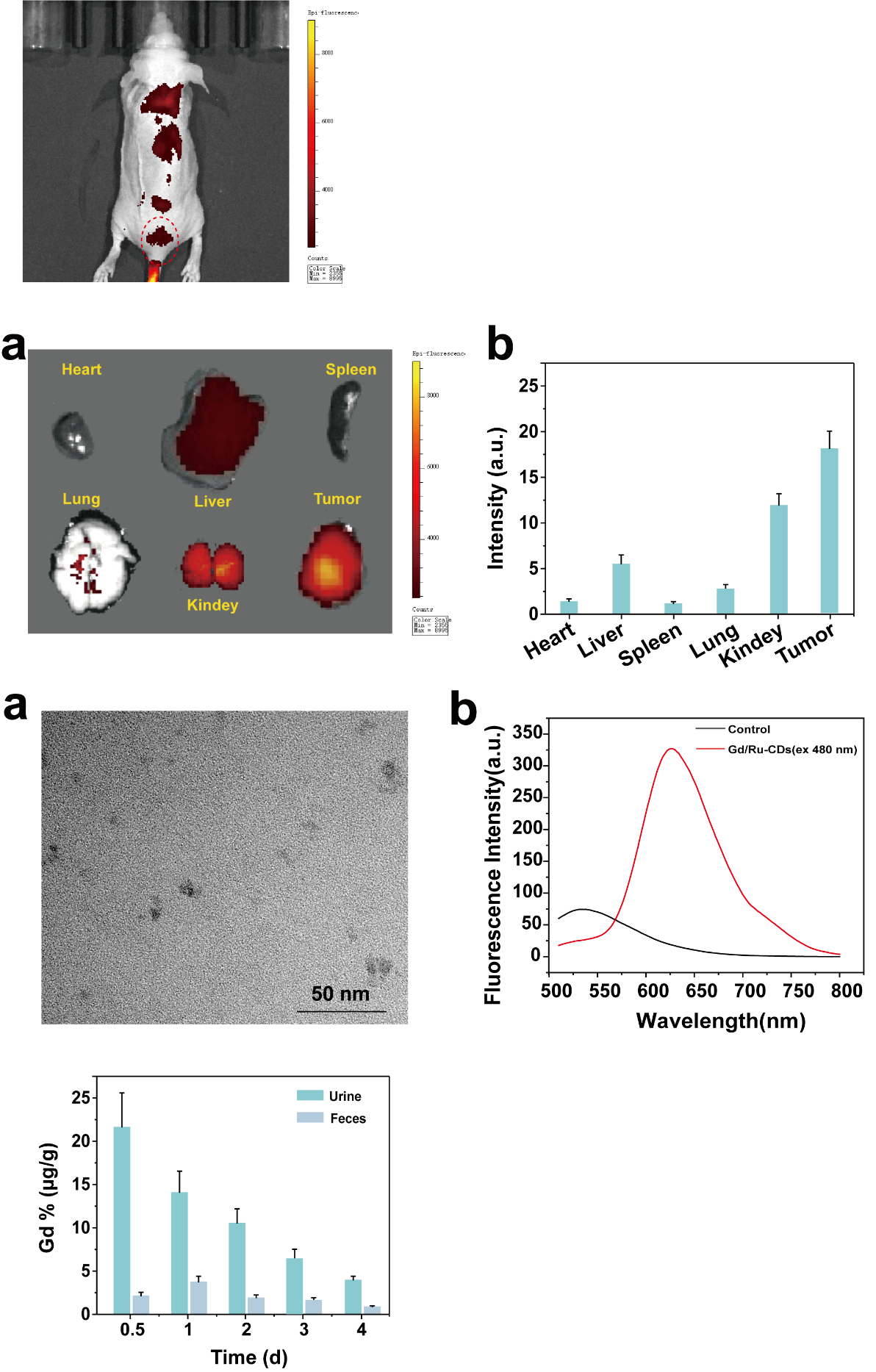


**Figure S14.** (a) Ex vivo fluorescence images of the major orangs and tumor tissue after injection of Gd/Ru-CDs at 4 h. (b) Column histograms of the relative fluorescence intensity in different organs obtained from the ex vivo imaging data.


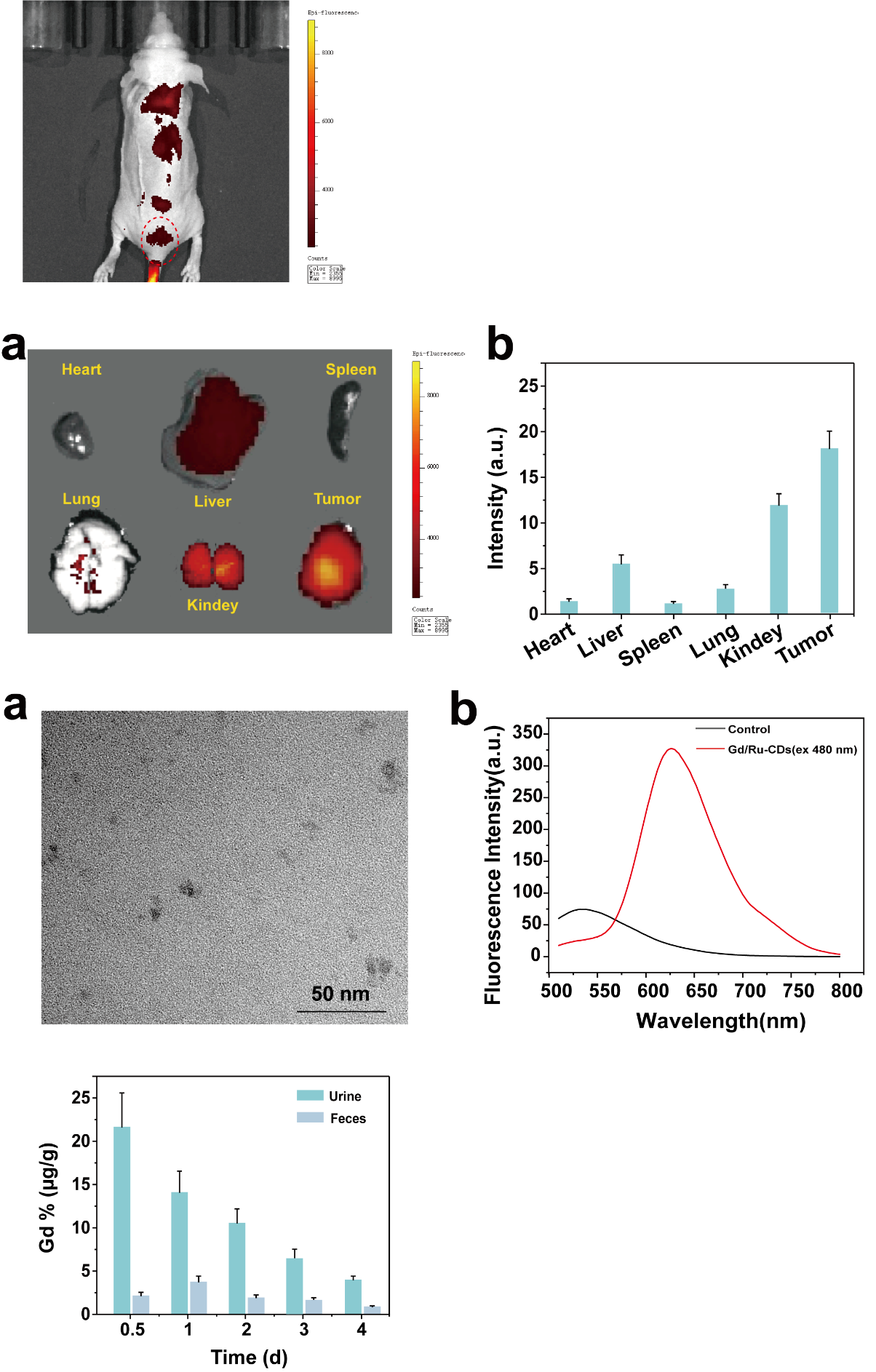


**Figure S15.** (a) TEM image of purified urines. (b) Fluorescent analysis of the urine samples obtained from the mice with and without injection of Gd/Ru-CDs.


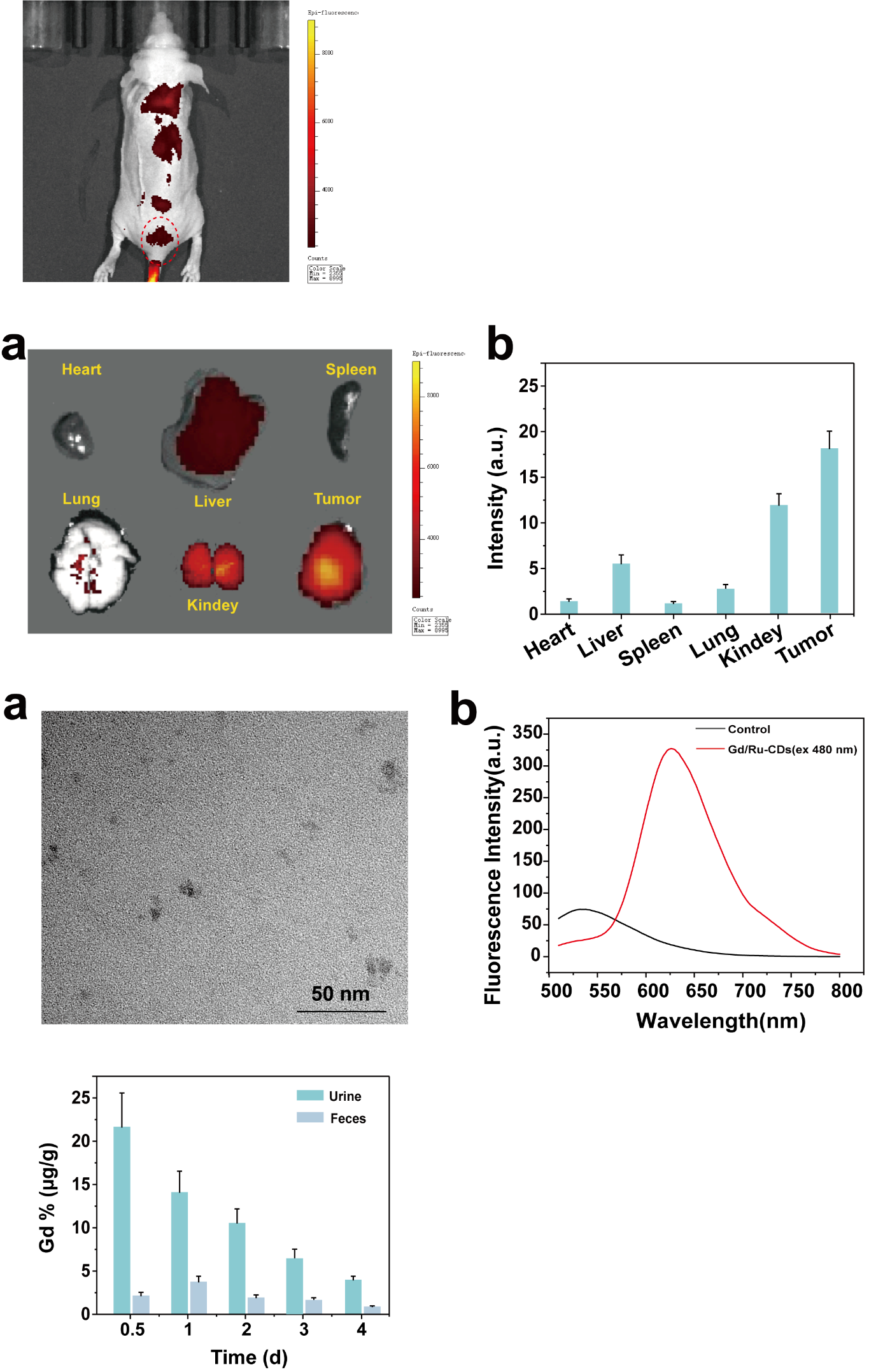


**Figure S16.** Gd content in collected urine and feces at various time intervals (0.5, 1, 2, 3, 4 days).
